# Virulence characteristics and antibiotic resistance analysis of *Klebsiella pneumoniae* isolated from pig farms in Xinjiang, China: revealing potential zoonotic risks

**DOI:** 10.1186/s40813-025-00424-x

**Published:** 2025-05-07

**Authors:** Sheng-Hui Wan, Nana Li, Pei Zheng, Yanfang Li, Yan Liang, Yonggang Qu

**Affiliations:** 1 College of Animal Science and Technology, Shihezi University, Shihezi, 832003 China; 2 Xinjiang Tecon Animal Husbandry Technology Co., Ltd., Changji, 831399 China

**Keywords:** Pigs, *K. pneumoniae*, Drug-resistant phenotype, Drug resistance gene, Virulence gene, Pathogenicity

## Abstract

**Background:**

This study aimed to analyze the antimicrobial resistance and pathogenicity of *Klebsiella pneumoniae*(*K. pneumoniae*) isolates from pigs, evaluate their potential threat to pig farming and public health, and provide a theoretical basis for controlling *K. pneumoniae* infections in pig farms.

**Methods:**

Nasal swabs collected from pigs were subjected to bacterial isolation, biochemical identification, species-specific PCR, and 16S rRNA sequencing to identify *K. pneumoniae*. Serotyping and multilocus sequence typing (MLST) were conducted using the *wzi* and MLST methods, respectively. Biofilm formation was assessed using crystal violet staining. Antimicrobial susceptibility was evaluated via the Kirby-Bauer disk diffusion method, and resistance and virulence genes were identified using PCR. Pathogenicity was determined through string testing and mouse infection models.

**Results:**

21 strains of *K. pneumoniae* were isolated and identified from 50 swabs of pig nasal cavities. The isolates were classified into serotypes wzi 19 and wzi 81 and sequence types ST37 and ST967. Ten isolates exhibited strong biofilm-forming ability, while 11 showed moderate biofilm production. Antimicrobial susceptibility testing revealed resistance to β-lactams, aminoglycosides, quinolones, tetracyclines, sulfonamides, aminoalcohols, and glycopeptides, with sensitivity restricted to imipenem and polymyxins. Ten resistance genes and eight virulence genes were detected. Pathogenicity testing in mice revealed a moderate virulence level, with a median lethal dose (LD_50_) of 4.0 × 10⁶ CFU/mL. Infected mice exhibited significant lesions in the liver, lungs, and small intestine.

**Conclusion:**

These findings highlight a potential risk to pig farming and public health, emphasizing the need for effective control measures against *K. pneumoniae* infections in pig farms.

**Supplementary Information:**

The online version contains supplementary material available at 10.1186/s40813-025-00424-x.

## Introduction

*K. pneumoniae* is a flagellate, encapsulated bacterium widely distributed in nature and is considered as an opportunistic pathogen that can cause diseases such as pneumonia, meningitis, septicemia, and bronchitis in humans and animals, especially when host immunity is compromised or after prolonged use of antimicrobial drugs. In pigs, *K. pneumoniae* infections commonly present as suppurative fibrinous pneumonia, mastitis, and septicemia [1, 2].

Several outbreaks of *K. pneumoniae* in pig farms have caused significant economic losses. Between 2011 and 2014, outbreaks in 13 UK pig farms led to septicemia and rapid mortality in piglets [3]. Similarly, in 2017, an outbreak in Victoria, Australia, resulted in a mortality rate of approximately 60% among piglets, severely affecting the pig farming industry [4]. Despite these high fatality rates, *K. pneumoniae* infections in animals remain underrecognized and under-researched.

The increasing scale of livestock farming, improper management practices, overuse of antibiotics, and prevalence of immunosuppressive diseases have contributed to a rise in antimicrobial-resistant *K. pneumoniae*. The emergence of multidrug-resistant (MDR) strains has exacerbated treatment challenges [5, 6]. Resistance in *K. pneumoniae* is often associated with transferable plasmids carrying both resistance and virulence genes, which enhance bacterial survival in adverse environments [7].

The pathogenicity of *K. pneumoniae* is primarily attributed to its four key virulence factors: capsular polysaccharides, pili, lipopolysaccharides (LPS), and siderophores. The polysaccharide capsule aids in evasion from host immune defenses, including phagocytosis, lysozyme, and complement-mediated damage. Of the 78 capsular serotypes identified, K1 and K2 are considered the most virulent [8]. Adhesins and type 1 (*fim*) and type 3 (*mrk*) pili facilitate bacterial adherence to epithelial and immune cells, promoting biofilm formation. LPS, composed of lipid A, core polysaccharide, and O antigen, functions as an immune activator and virulence factor. Lipid A, in particular, helps suppress host inflammatory responses, while the diversity of O antigens enables immune evasion [9, 10].

Siderophores, including enterobactin, salmochelin, yersiniabactin, and aerobactin, allow *K. pneumoniae* to thrive in iron-limited environments by outcompeting host iron-chelating proteins [11]. Additional virulence factors, such as hemolysin and enterotoxin, further contribute to its pathogenic potential [12].

In this study, 21 strains of *K. pneumoniae* were isolated from nasopharyngeal swabs collected at a large-scale pig farm in Xinjiang, China. The isolates were analyzed for antimicrobial resistance, pathogenicity, and the presence of resistance and virulence genes. These findings aim to inform precise treatment strategies for *K. pneumoniae* infections in pig populations and highlight potential public health risks.

## Materials and methods

### Animal sampling and testing

#### Sampling

**Fig. 1:**
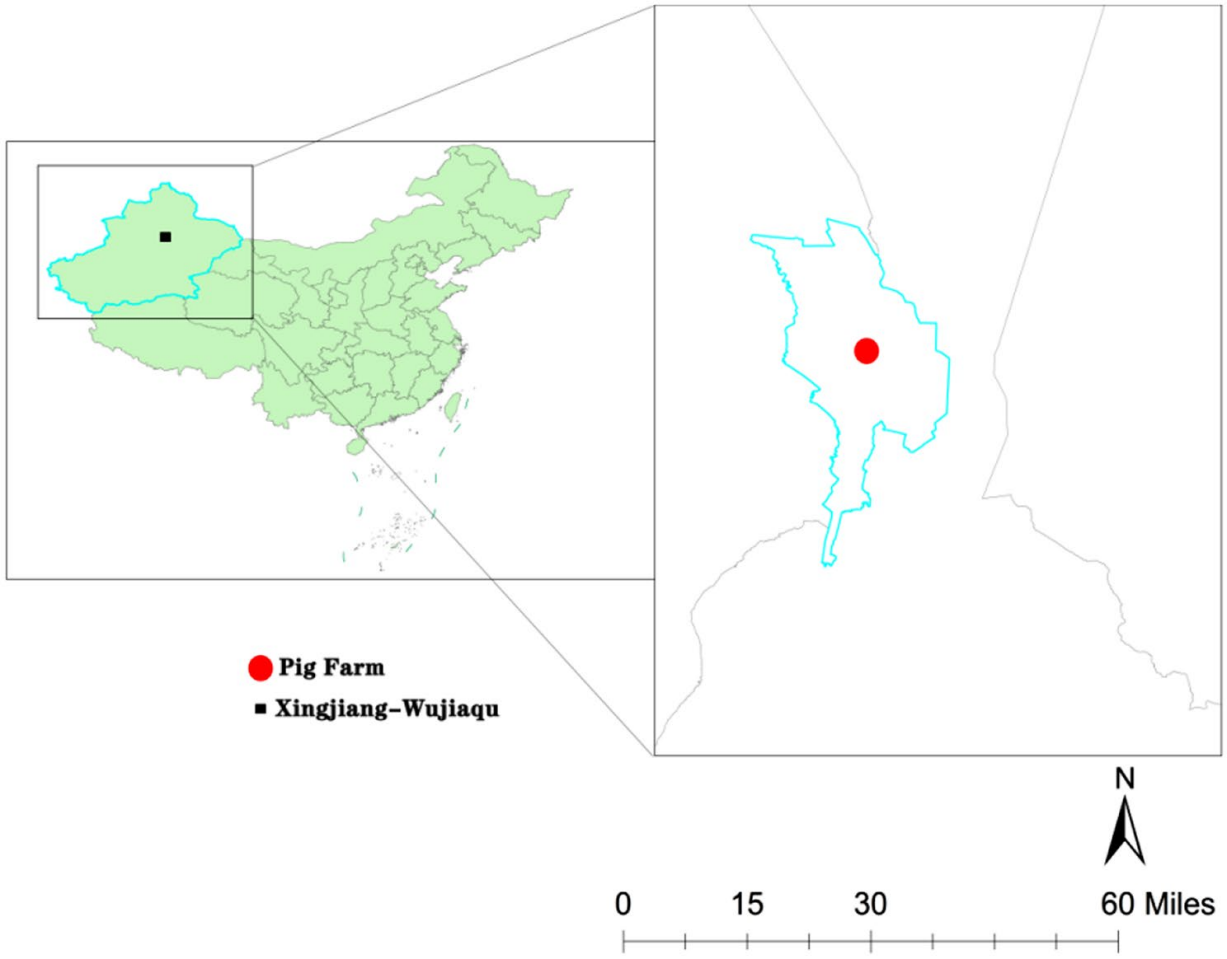
Geographical distribution of sampling sites in Xinjiang

From May to August 2024, we choosed Wujiaqu, a city situated in central Xinjiang, renowned for its numerous pig farms, as the geographical area for our research, and collected 50 nasal swabs from the largest pig farm with 15,000 hybrid pig (Duroc × Landrace × Yorkshire). From 5 pig pens, we collected nasal swabs from 50 healthy hybrid piglets, aged 30 days and weighing (10 ± 0.5) kg. The nasal swabs were collected by inserting them into the animals’ nasal cavities and rotating them 2–3 times. The samples were immediately stored at 4 °C for subsequent analysis. The swine farm is located in the central part of Xinjiang, which has developed transportation (Fig. 1). At the same time, the management of the swine farm is strict, and it can sell a large number of piglets and pork products to the outside world.

#### Experimental animals

A cohort of 48 six-week-old Kunming strain white mice was procured from the Laboratory Animal Center of Xinjiang Medical University. The mice were used for pathogenicity assays under controlled laboratory conditions (Approval Letter for Ethics Review by Biology Ethics Committee of Shihezi University, Approval Number: A2024-412).

#### Isolation and purification of *K. pneumoniae*

Nasal swabs from pigs were inoculated into brain heart infusion (BHI) liquid medium (Haibo Biotech, Qingdao, China) under aseptic conditions and incubated overnight at 37 °C with shaking at 180 rpm. The enriched cultures were then streaked onto MacConkey agar plates (Haibo Biotech, Qingdao, China) and incubated at 37 °C for 24 h.

Single colonies were selected and repeatedly streaked onto fresh MacConkey agar plates for purification until uniform colony size and morphology were achieved. Colony characteristics, including size, morphology, and color, were recorded. Gram-staining (Solarbio Technology, Beijing, China) was performed for microscopic examination to confirm bacterial morphology and Gram reaction.

### Identification of *K. pneumoniae*

#### Biochemical identification

The isolated strains were identified using a biochemical identification tube kit (Hangzhou Microbial Reagent Co., Hangzhou, China) according to the manufacturer’s instructions.

#### PCR identification and 16 S rRNA gene genetic evolution analysis

The genomic DNA of bacterial isolates was extracted by boiling methods as template. PCR detection was performed using specific primers designed for the *Khe* gene unique to *K. pneumoniae*, with the genomic DNA of bacterial isolates serving as the template. PCR program: 3 min pre-denaturation at 95 °C; 25s denaturation at 94 °C; 25s annealing at 61 °C (specific annealing temperature see Table 1); 1 min extension at 72 °C, for a total of 30 cycles; 10 min extension at 72 °C. Verify PCR products using 1% agarose gel electrophoresis. Using universal bacterial primers [13] to amplify the 16 S rRNA gene of the isolated strains, the purified PCR products were sent to General Biologicals Co., Ltd. (Anhui, China) for sequencing. The assembled complete sequences were subjected to BLAST comparison on NCBI, and a phylogenetic tree was constructed using MEGA 11.0. The primers were synthesized by Xinjiang Youkang Biotechnology Co., Ltd. (Urumqi, China), and the primer sequences are shown in Table 1.

**Table 1:** Primer sequences for identification of *K. pneumoniae*

Primer name	Primer sequence (5 ‘-3’)	Fragment size (bp)	Annealing temperature (°C)
*Khe*	F: ACAGCCCGGAGCGTTTTTC	285 bp	61
	R: ACCACCAGCAGACGAACYTCC		
16 S rRNA	F: AGAGTTTGATCCTGGCTCAG	1 476 bp	58
	R: TACGGCTACCTTGTTACGACTT		

#### Capsular serotyping and MLST analysis

The *wzi* gene sequencing method was used to determine the capsular polysaccharide type of the isolates, while the MLST method was employed to identify the sequence type (ST) of the isolates as described in [14], primers for the *wzi* gene of *K. pneumoniae* and seven housekeeping genes (*gapA*,* infB*,* mdh*,* pgi*,* phoE*,* ropB*, and *tonB*) were designed and subjected to PCR amplification. The primer sequences and related information are detailed in Table 2. PCR reaction program: 95 °C for 3 min for pre-denaturation; 94 °C for 25s for denaturation; 60 °C for 25s for annealing; 72 °C for 1 min for extension, set for 30 cycles; followed by a final extension at 72 °C for 5 min. Use 1% agarose gel electrophoresis to validate the PCR products. The PCR positive products were sequenced using the Sanger method, with the sequencing performed by General Biologicals (Anhui, China). Submit the *wzi* gene sequence to the online (https://bigsdb.pasteur.fr/klebsiella/). The capsular serotypes of the isolates were obtained, and 7 housekeeping gene sequences were uploaded to MLST network database, and the ST types of the isolates were obtained.

**Table 2:** *K. pneumoniae* typing identification primer sequence

Gene name	Primer sequence (5 ‘-3’)	Fragment size (bp)	Annealing temperature (°C)
*ropB*	F: GGCGAAATGGCWGAGAACCA R: GAGTCTTCGAAGTTGTAACC	1075	50
*gapA*	F: TGAAATATGACTCCACTCACGG R: CTTCAGAAGCGGCTTTGATGGCTT	662	60
*mdh*	F: CCCAACTCGCTTCAGGTTCAG R: CCGTTTTTCCCCAGCAGCAG	756	50
*pgi*	F: GAGAAAAACCTGCCTGTACTGCTGGC R: CGCGCCACGCTTTATAGCGGTTAAT	566	50
*phoE*	F: ACCTACCGCAACACCGACTTCTTCGG R: TGATCAGAACTGGTAGGTGAT	602	50
*infB*	F: CTCGCTGCTGGACTATATTCG R: CGCTTTCAGCTCAAGAACTTC	462	50
*tonB*	F: CTTTATACCTCGGTACATCAGGTT R: ATTCGCCGGCTGRGCRGAGAG	539	45
*wzi*	F: GTGCCGCGAGCGCTTTCTATCTTGGTATTCC R: GAGAGCCACTGGTTCCAGAACTTGACCGC	580	60

#### Quantitative biofilm production assay

The ability of *K. pneumoniae* to form biofilm was determined by crystal violet staining. *K. pneumoniae* isolates were inoculated with LB solid medium by four-zone method and cultured at 37 °C for 12 h. A single colony was selected and inoculated in LB liquid medium, and cultured at 37 °C and 180 r/min until the concentration of the liquid reached 0.5 mL. 200 µL of the culture medium was transferred to the sterile 96-well plate and incubated at 37 °C for 48 h. After culture, the medium was discarded, washed 3 times with PBS buffer, and fixed with methanol solution (200 µL/Well) for 15 min. PBS buffer was washed three times, 96-well cell plates were naturally dried and then stained with 0.1% crystal violet staining solution (200 µL/Well) for 10 min at room temperature, and a large amount of sterile distilled water was used for multiple washings until colorless, after decolorization with anhydrous ethanol (200 µL/well), the absorbance at 570 nm was measured by Enzyme-linked Immunosorbent Assay Reader. The critical ODc values (ODc = the mean of the blank control wells) were used to determine the results: OD ≤ ODc was considered to have no ability to form membrane, ODc < OD ≤ 2ODc was considered to have weak ability to form membrane, and 2ODc < OD ≤ 4ODc was considered to have moderate ability to form membrane, OD > 4ODc is the strong ability of film formation.

#### Antimicrobial susceptibility testing

The paper disc diffusion method (Kirby-Bauer method) was used to assess the sensitivity of isolated strains to common antibiotics. A 200µL aliquot of the logarithmic growth-phase culture of *K. pneumoniae* was uniformly spread on a Mueller-Hinton agar (MHA) plate. Discs containing 17 different antibiotics(PEN, AMX, CFB, CTX, K, GM, ENR, CIP, DX, TMP, FON, PB, AMP, IPM, TCY, CRO, CA) were placed at appropriate positions on the plate, which was then incubated at 37 °C for 24 h. The diameters of the inhibition zones were measured and recorded, with the experiment repeated three times to obtain an average value. Concurrently, *K. pneumoniae* ATCC 700,603 and *Escherichia coli* ATCC 25,922 were used as quality control strains for parallel testing to ensure the validity of the results. The sensitivity of the bacteria to the antibiotics was determined based on the guidelines from the Clinical and Laboratory Standards Institute (CLSI) [15– 16] (Table 3).

**Table 3:** Criteria for determining the dosage and drug resistance of tablets

Antibiotic name	Drug content (µg/tablet)	Criteria for determining the diameter of the antibacterial zone/ mm		
		*R*	I	S
PEN	10 U	≤ 13	14–16	≥ 17
AMX	10	≤ 13	14–16	≥ 17
CFB	30	≤ 19	20–22	≥ 23
K	30	≤ 13	14–17	≥ 18
GM	10	≤ 12	13–15	≥ 16
ENR	5	≤ 13	14–16	≥ 17
CIP	5	≤ 21	22–25	≥ 26
DX	30	≤ 10	11–13	≥ 14
TMP	23.75/1.25	≤ 10	11–15	≥ 16
FON	30	≤ 12	13–16	≥ 17
PB	30 IU	≤ 8	9–11	≥ 12
CTX	30	≤ 22	23–25	≥ 26
AMP	10	≤ 13	14–16	≥ 17
IPM	10	≤ 19	20–22	≥ 23
TCY	30	≤ 11	12–14	≥ 15
CRO	30	≤ 19	20–22	≥ 23
CA	30	≤ 14	15–16	≥ 17

#### Antimicrobial resistance gene detection

Referring to relevant literature [17–21], Using PCR methods to detect six categories of 16 antibiotic resistance genes in isolates of *K. pneumoniae*, which include β-lactam (*bla*_*TEM*_, *bla*_*CTX*_, *bla*_*NDM*_, *bla*_*OXA−48*_, *bla*_*IMP*_, *bla*_*VI*_, *bla*_*DHA*_, *bla*_*KPC*_), aminoglycosides (*aadA*), tetracyclines (*tet(B)*), chloramphenicol (*cmlA*,* floR*), sulfonamides (*sul2*), and quinolones (*parC*,* gyrA*,* gyrB*). The PCR reaction conditions are the same as described in Sect. 2.3.2, with specific primer information detailed in Table 4.

**Table 4:** Primer sequences for resistance genes of *K. pneumoniae*

Types of antibacterial drugs	Gene name	Primer sequence (5 ‘-3’)	Fragment size (bp)	Annealing temperature (°C)
β - lactams	*bla*_*TEM*_	F: TCGCCGCATACACTATTCTCAGAATGA R: ACGCTCACCGGCTCCAGATTTAT	445	55
	*bla*_*CTX*_	F: ACTTCAGCCACACGGATTCA R: AAGTGGAGCGACAGAGC	905	57
	*bla*_*NDM*_	F: GGTTTGGCGATCTGGTTTTC R: CGGAATGGCTCATCACGATC	621	55
	*bla*_*OXA−48*_	F: GCGTGGTTAAGGATGAACAC R: CATCAAGTTCAACCCAACCG	438	55
	*bla*_*IMP*_	F: GGAATAGAGTGGCTTAAYTCTC R: GGTTTAAYAAAACAACCACC	232	55
	*bla*_*VIM*_	F: GATGGTGTTTGGTCGCATA R: CGAATGCGCAGCACCAG	390	55
	*bla*_*DHA*_	F: GCCTGTTTGGTGCTCTGA R: GCACGGTTATACGGCTGA	460	55
	*bla*_*KPC*_	F: CGTCTAGTTCTGCTGTCTTG R: CTTGTCATCCTTGTTAGGCG	798	55
Aminoglycosides	*aadA*	F: GTGGATGGCGGCCTGAAGCC R: AATGCCCAGTCGGCAGCG	525	62
Tetracyclines	*tet(B)*	F: AAACCATTACGGCATTCTGC R: GACCGGATACACCATCCATC	659	55
Chloramphenicol class	*cmlA*	F: GGCCTCGCTCTTACGTCATC R: GCGACACCAATACCCACTAGC	989	57
	*floR*	F: TATCTCCCTGTCGTTCCAG R: AGAACTCGCCGATCAATG	399	53
Sulfonamide class	*sul2*	F: CGGCATCGTCAACATAACCT R: TGTGCGGATGAAGTCAGCTC	721	66
Quinolones	*parC*	F: CTGGGTAAATACCATCCGCAC R: CGGTTCATCTTCATTACGAA	987	59
	*gyrA*	F: ATGAGCGACCCTTGCGAGAGAAAT R: AGCCCTTCAATGCTGATGTCTTC	685	60
	*gyrB*	F: CCTCCCAGACCAAAGACAAACT R: CAGCATTGCTTTCGGATAACG	821	53

#### String test

*K. pneumoniae* isolates were streaked onto MacConkey agar plates and incubated at 37 °C for 12 h. Using an inoculation loop, a single colony was gently touched and slowly lifted to observe the formation of a string-like growth.

The length of the slime string was measured and recorded. Isolates were classified as mucoid if they produced a string and as hypermucoviscous (HV) if the string length exceeded 5 mm [22].

#### Virulence gene detection

Referring to relevant literature [18, 23–27], the PCR method was employed to detect 12 virulence genes in *K. pneumoniae* strains: lipopolysaccharide-related genes (*uge*,* wabG*), fimbriae-related genes (*fimH*,* mrkD*), iron transport-related genes (*entB*,* kfu*,* iroN*,* icuA*), urease-related gene (*ureA*), and allantoin-related gene (*allS*). The PCR reaction system and procedure were consistent with Sect. 2.3.2, and the primer sequences and related information are provided in Table 5.

**Table 5:** Primer sequences for virulence genes of *K. pneumoniae*

Toxicity gene types	Gene name	Primer sequence (5 ‘-3’)	Fragment size (bp)	Annealing temperature (°C)
Pili	*fimH*	F: GTCTACGTTAACCTGACCCCG R: ATTGATAGACAAAGGTGATGCCGAT	781	56
	*mrkD*	F: AAGCTATCGCTGTACTTCCGGCA R: GGCGTTGGCGCTCAGATAGG	340	60
lipopolysaccharide	*uge*	F: TCTTCACGCCTTCCTTCACT R: GATCATCCGGTCTCCCTGT	534	53
	*wabG*	F: CTCTGGTGCGGCAGAAGTAC R: TGGCCGTCGACGATAAACTC	931	52
Capsule	*rmpA*	F: ACCCTTTACAGCCAAATTTTCTTGT R: CTGGGCTACCTCTGCTTCATAT	468	52
	*magA*	F: TGATAAGTGGCGGAGATTCTGA R: TGATAAGTGGCGGAGATTCTGA	542	52
	*iroN*	F: GAATGAAACTACCGCCCCCA R: TGTGGAGTGGAGGCGAGATA	1 033	54
Iron carrier	*entB*	F: ATTTCCTCAACTTCTGGGGC R: AGCATCGGTGGCGGTGGTCA	371	60
	*iucA*	F: GCTTATTTCTCCCCAACCC R: TCAGCCCTTTAGCGACAAG	583	59
	*kfu*	F: GAAGTGACGCTGTTTCTGGC R: TTTCGTGTGGCCAGTGACTC	797	55
Allantoin	*allS*	F: CTTCAGCAGATAAATGACGGGGTAG R: GTGGGTAAACCGCCATATTTTCC	244	56
urease	*ureA*	F: GCTGACTTAAGAGAACGTTATG R: GATCATGGCGCTACCT(C/T)A	337	55

#### Mouse pathogenicity test

Based on the detection of resistance and virulence genes, a multidrug-resistant strain, KP-20, belonging to ST 967 and carrying eight virulence genes and seven resistance genes, was selected as the challenge strain.

### Experimental design

A total of 48 six-week-old Kunming strain mice were randomly assigned to eight groups (one control group and seven experimental groups), with six mice per group. Prior to the experiment, the mice were fasted but allowed access to water for 24 h.A fresh bacterial culture in the logarithmic growth phase was prepared and subjected to a 10-fold gradient dilution. The experimental group of mice was intraperitoneally injected with 0.4 mL of bacterial suspensions at various doses ranging from 1.6 × 10⁹ to 1.6 × 10³ CFU per mouse. The control group was injected with an equal volume of sterile saline.

### Observation and data collection

Post-infection, the clinical symptoms and mortality of the mice were observed and recorded every four hours. The median lethal dose (LD₅₀) of KP-20 was calculated using the modified Karber method.

### Necropsy and pathological examination

Mice that succumbed to the infection underwent necropsy to assess organ lesions. Diseased tissues were collected for bacterial isolation and culture. Isolates were identified as *K. pneumoniae* using specific primers. Additionally, affected tissues were fixed in 4% formaldehyde, embedded in paraffin, sectioned, and stained for histopathological examination under a microscope.

#### Statistical analysis

All statistical analyses were performed using GraphPad Prism 9.0,Graphs and charts were generated to visually represent the statistical findings, aiding in the interpretation of the results.

## Results

### Isolation and identification of *K. pneumoniae*

**Fig. 2:**
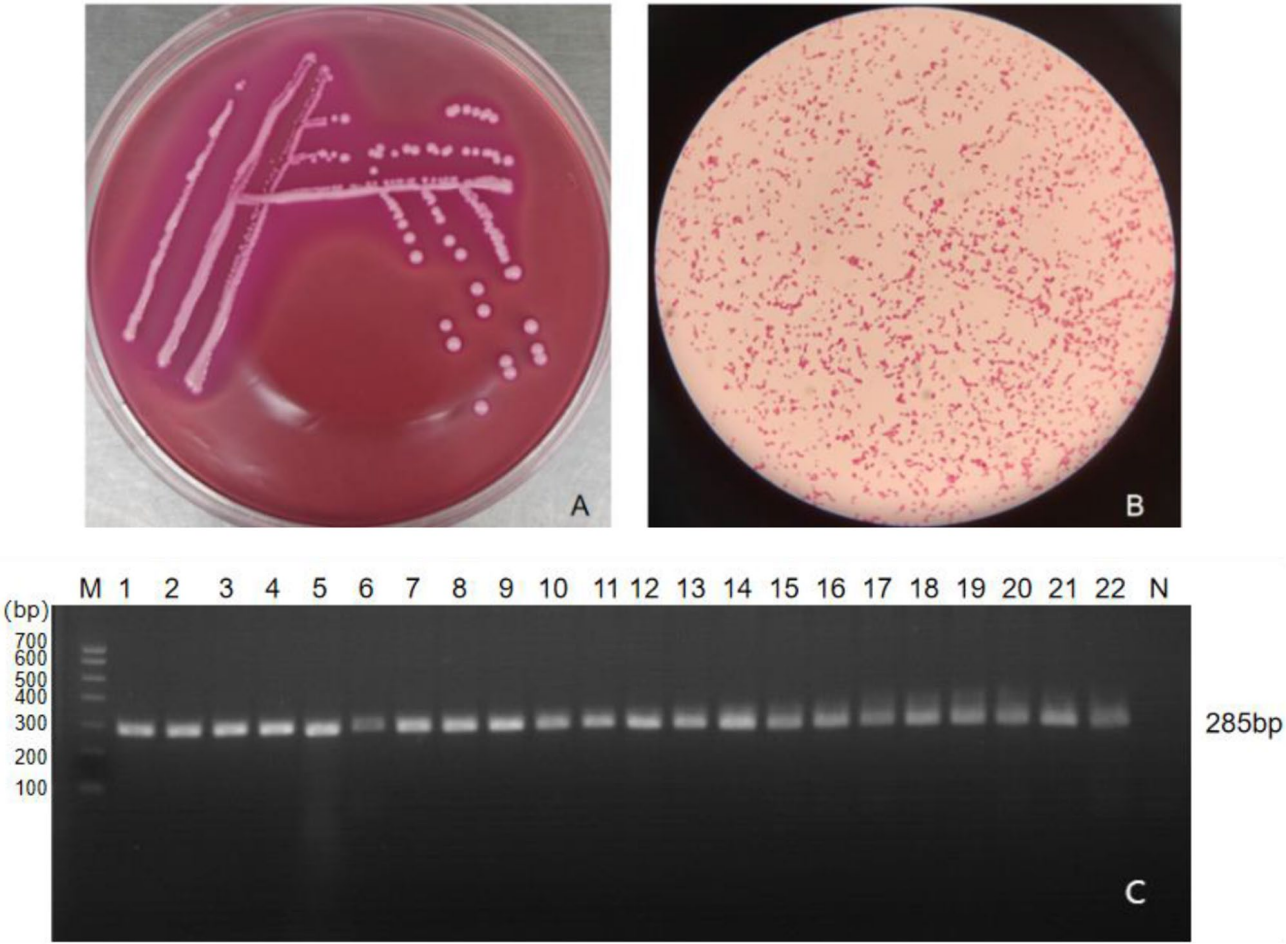
Identification of isolated strains. *Note*** A** Colony morphology of isolated bacteria on MacConkey agar medium; **B** Gram-staining microscopy results of isolated bacteria (100x); **C** PCR amplification results of *Khe* gene of isolated bacteria (M: DL-700 Marker 1–22: Isolate Strain N: Negative Control)

A total of 21 bacterial strains were isolated from the collected samples. The isolated strains grew as pink, mucoid, circular colonies on MacConkey agar (Fig. 2A). Gram-staining microscopy reveals the presence of single, paired, or chained, blunt-ended, pink short bacilli (see Fig. 2B). Biochemical characteristic analysis shows that the isolated strain tests positive for glucuronate, peptone water, citrate, gas production from glucose, lysine, gossypol, sorbitol, marigold alcohol, and xylose. Negative reactions are observed for hydrogen sulfide, indole, phenylalanine, semi-solid, and ornithine tests. The biochemical characteristics of the isolated bacterium are consistent with those of *K. pneumoniae* as described in the Bergy’s Manual of Determinative Bacteriology. PCR identification of the isolated strain was performed using *Khe* gene primers specific to *K. pneumoniae*, with the PCR amplification product showing a specific band at 285 bp, consistent with the expected fragment size (see Fig. 2C).

**Fig. 3:**
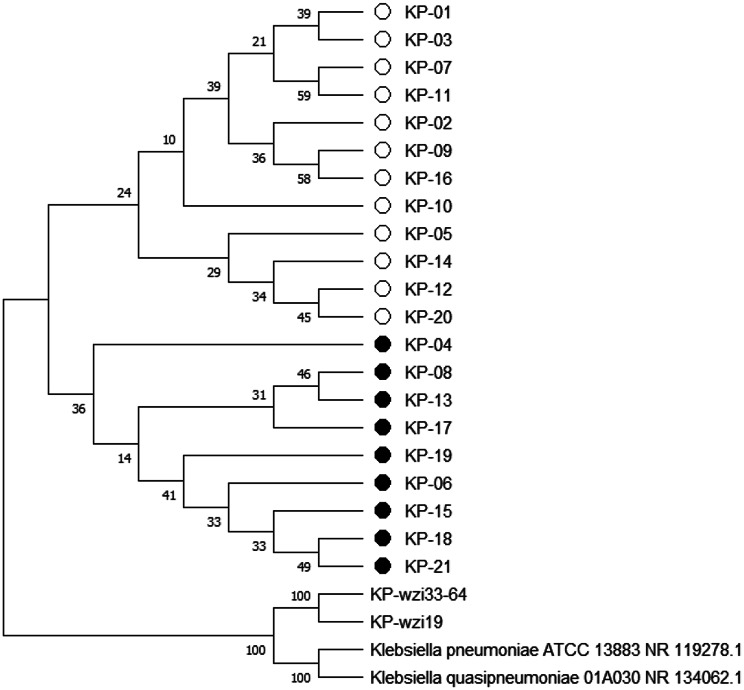
Phylogenetic tree based on 16 S rRNA gene sequence. *Note* The phylogenetic tree of 16 S rRNA gene sequence of isolated strains (the strains marked with circles are all *K. pneumoniae* isolated in this study, and the hollow circles represent type ST 967; The solid circle represents the ST 37

Through the homology analysis of the 16 S rRNA gene, it was found that the 16 S rRNA gene sequences of 21 isolated strains exhibited a similarity of 99.86–100% with the 16 S rRNA sequences of *K. pneumoniae* in the GenBank database, further confirming that these isolates are indeed *K. pneumoniae*. Phylogenetic analysis indicated that the isolates mainly clustered into two branches, with the closest genetic relationship to the pig-derived *K. pneumoniae* previously isolated from the Yili region of Xinjiang [26] (KP-wzi19 and KP-wzi33-64) (see Fig. 3). Based on the biological characteristics of the isolates, the PCR amplification results of the *Khe* gene, and the analysis of the 16 S rRNA sequences, all 21 isolates were identified as *K. pneumoniae* and were designated as *KP*-01, *KP*-02, *KP*-03, *KP*-21. Among the 50 nasal swab samples, the overall isolation rate of *K. pneumoniae* was 42% (21/50).

#### Capsular serotyping and multilocus sequence typing

**Fig. 4:**
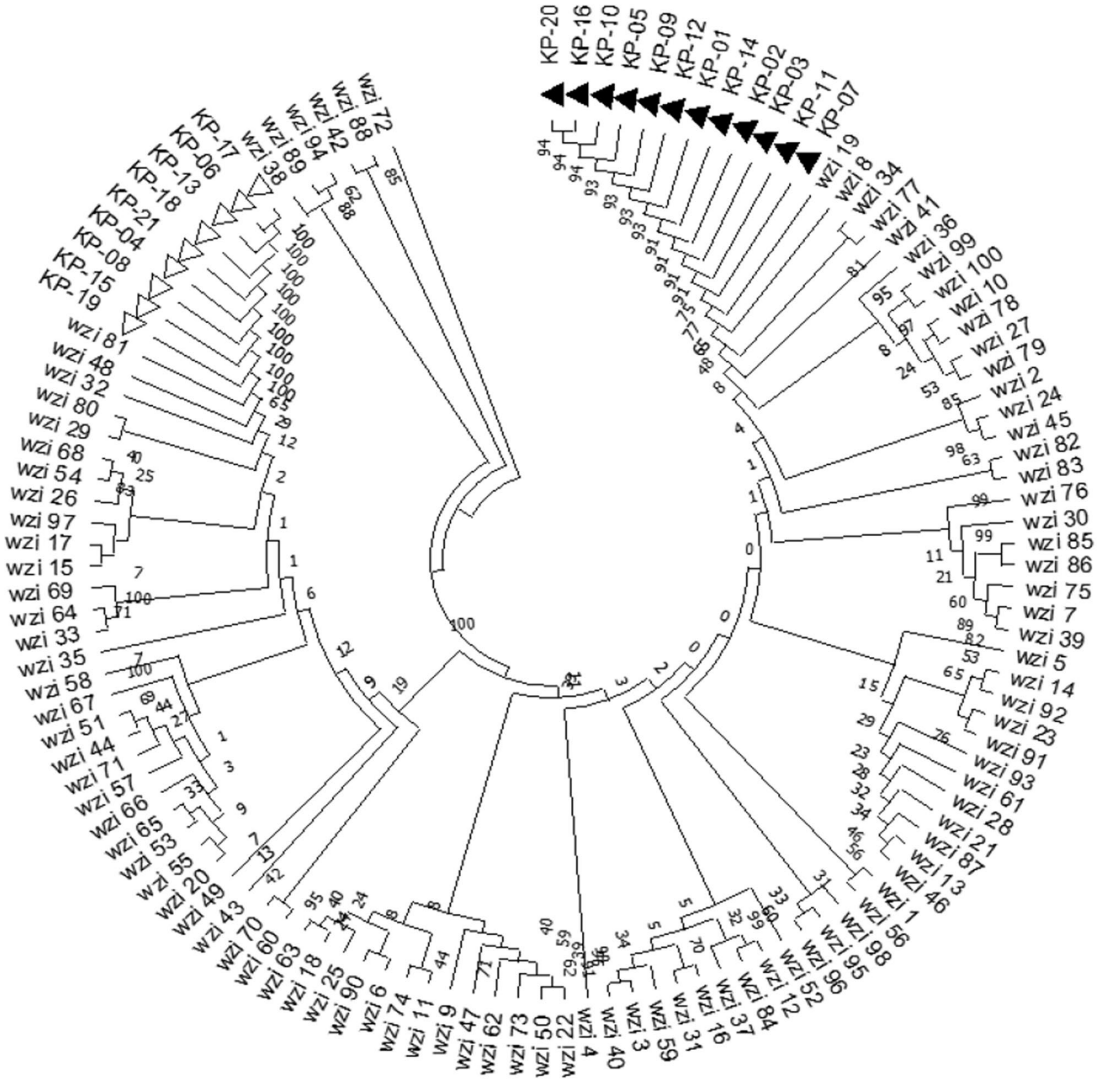
Identification of isolated strain serotypes

**Fig. 5:**
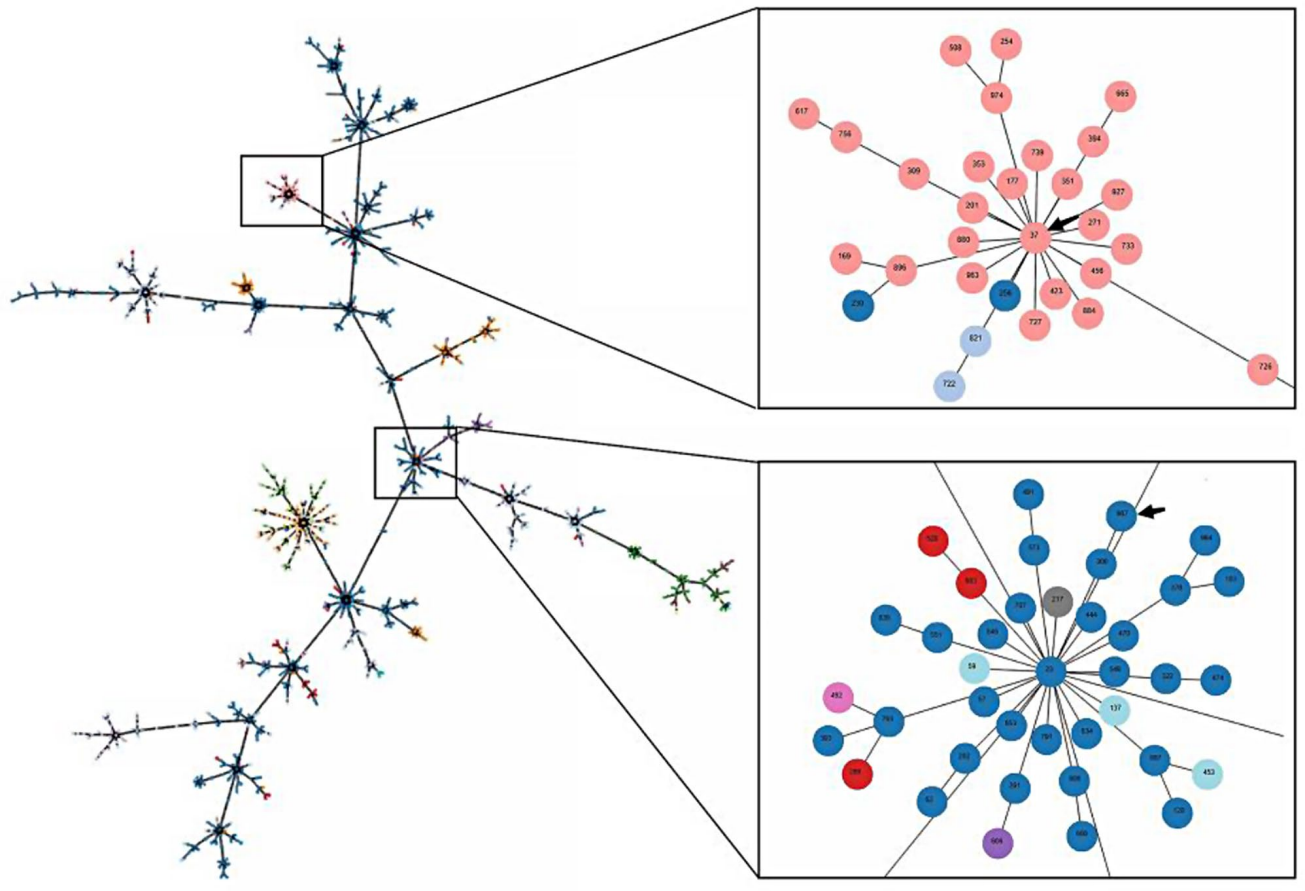
MLST minimum spanning tree

Based on the *wzi* gene sequence, 21 isolates of *K. pneumoniae* can be classified into two different *wzi* allele types. Among these, the wzi81-KL81KL120-K81 type accounts for 42.86% (9/21), while the wzi19-KL19-K19 type has the highest representation at 57.14% (12/21). The evolutionary tree of the *wzi* gene is shown in Fig. 4. According to 7 butler gene sequences, 21 strains of *K. pneumoniae* can be divided into two STs types, mainly ST 37 and ST 967. Among them, ST 37 accounted for 42.86 (9/21), and ST 967 accounted for 57.14% (12/21). The MLST minimum spanning tree found that the isolates ST 37 and ST 967 were far apart from each other (as shown in Fig. 5). The *K. pneumoniae* isolates of wzi81 belong to ST 37, and the isolates of wzi19 belong to ST 967, indicating that the two types of typing results are highly consistent. The detailed typing of *K. pneumoniae* isolates is shown in Table 6.

**Table 6:** Classification of *K. pneumoniae* isolates is shown

Strain name	wzi allele types	ST
KP-04	81	37
KP-06	81	37
KP-08	81	37
KP-13	81	37
KP-15	81	37
KP-17	81	37
KP-18	81	37
KP-19	81	37
KP-21	81	37
KP-01	19	967
KP-02	19	967
KP-03	19	967
KP-05	19	967
KP-07	19	967
KP-09	19	967
KP-10	19	967
KP-11	19	967
KP-12	19	967
KP-14	19	967
KP-16	19	967
KP-20	19	967

#### Biofilm production

**Fig. 6:**
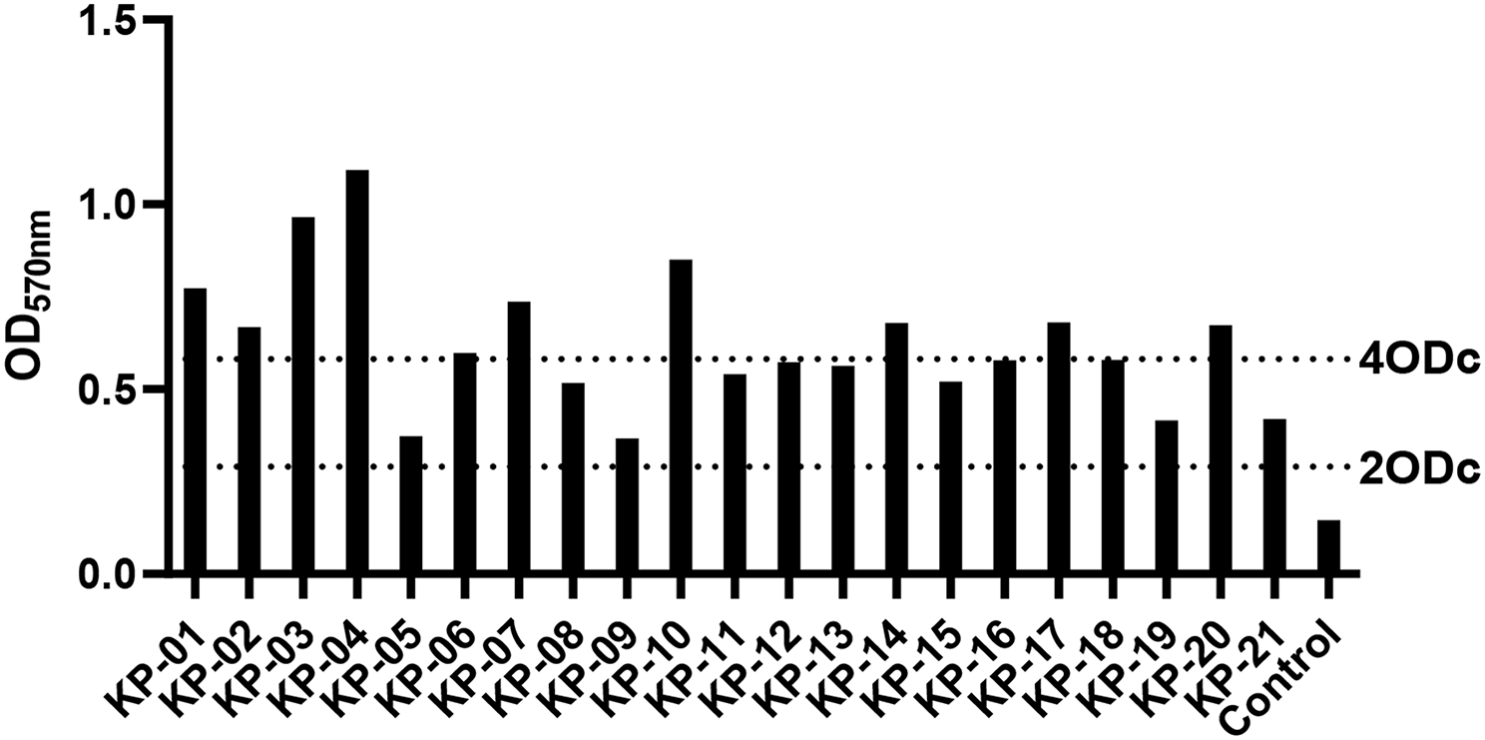
Isolate the biofilm formation ability of the strain

According to crystal violet staining method, OD_570nm_ of 21 isolates were all greater than 2 ODc (as shown in Fig. 6), among which 10 had strong film forming ability and 11 had medium film forming ability. There were 3 strains with strong film forming ability and 6 strains with medium film forming ability of type ST 37. There were 7 strains with strong film forming ability and 5 strains with medium film forming ability of type ST 967. It is suggested that these strains have strong ability to resist the external environment and the bactericidal effect of antibiotics.

#### Antimicrobial susceptibility testing

**Fig. 7:**
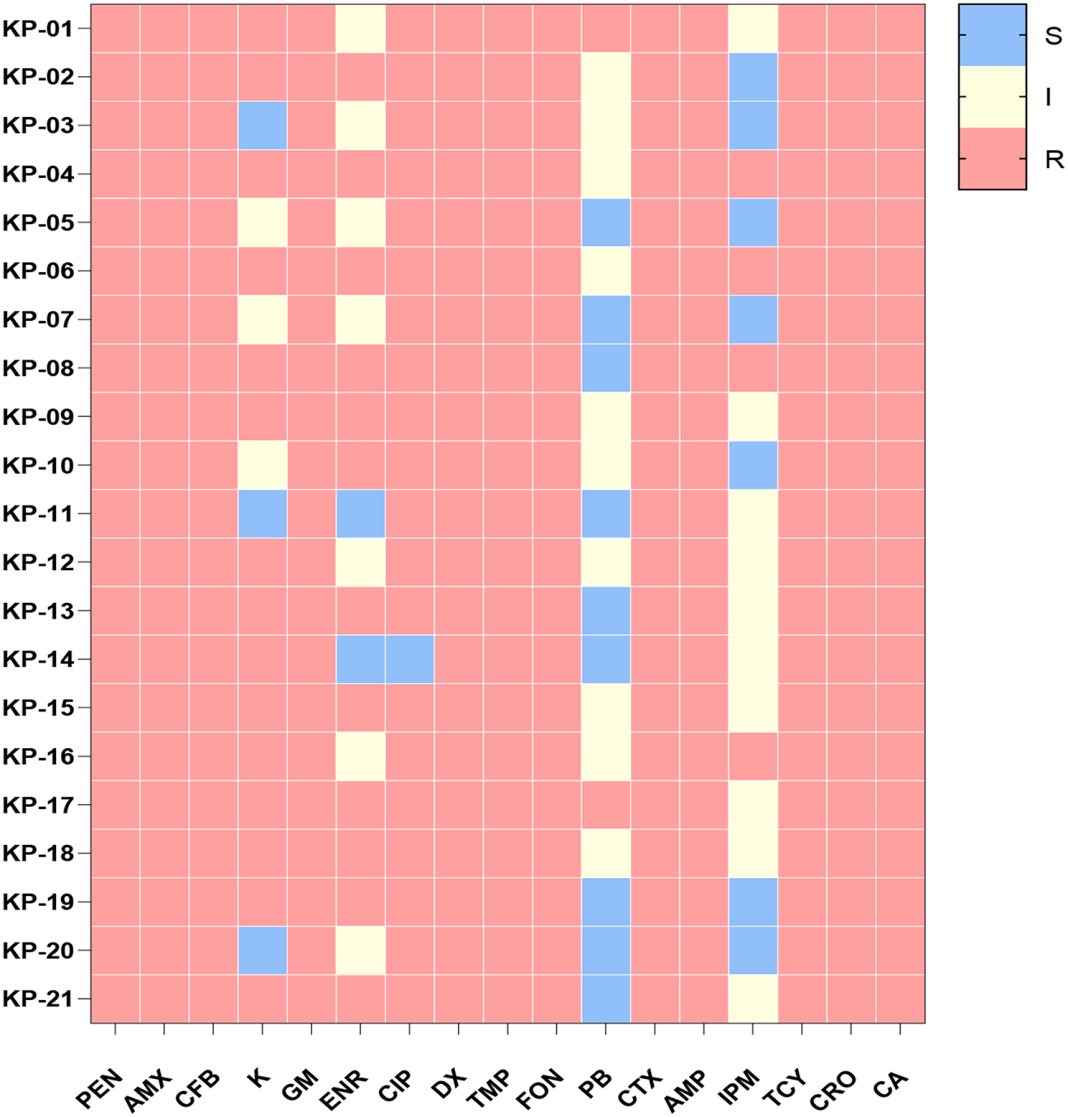
Overall durg resistance of 21 strains of *K. pneumoniae* to different types of antibiotics

The drug susceptibility test results of *K. pneumoniae* isolates were shown in Fig. 7. The 21 isolates showed strong resistance to β-lactam antibiotics and tetracycline antibiotics. Among them, penicillin, amoxicillin, cefazolin, ampicillin, cefotaxime, ceftriaxone for β-lactam antibiotics; Aminoglycoside gentamicin; Tetracycline doxycycline, flufenicol, tetracycline; Cotrimoxazole sulfanilamide; The drug resistance rate of 12 antibiotics such as glycopeptide vancomycin was up to 100%. The resistance rate of kanamycin to aminoglycoside was 71.43%. The resistance rates to Enrofloxacin and ciprofloxacin quinolones were 57.14% and 95.24%, respectively. These isolates were sensitive to carbapenem imipenem and polymyxin B, with resistance rates of 19.05% and 9.21% respectively. Each *K. pneumoniae* strain is resistant to at least 12 antibiotics, and according to the definition of bacterial multi-drug resistance (MDR), all isolates belong to the MDR strain.

#### Antimicrobial resistance genes

**Fig. 8:**
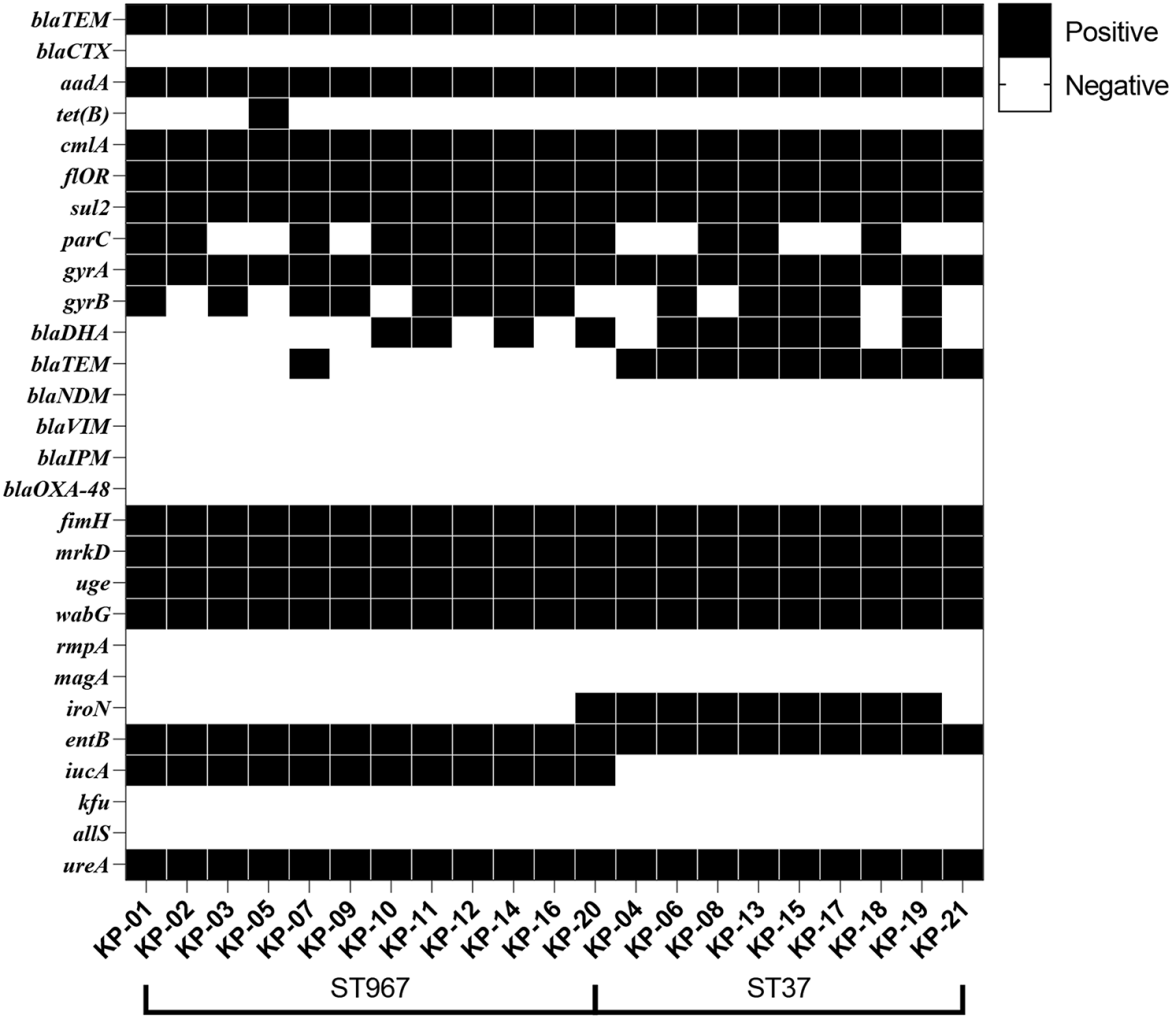
Detection of virulence genes in 21 strains of *K. pneumoniae*

The *K. pneumoniae* isolates were tested for 16 kinds of drug resistance genes, and the results were shown in Fig. 8. A total of 10 drug resistance genes in 6 classes (*bla*_*TEM*_, *bla*_*DHA*_, *aadA*,* cmlA*,* floR*,* sul2*,* tet(B)*,* parC*,* gyrA*,* gyrB*) were detected in 21 isolates. The detection rates of aminoglycoside resistance gene (*aadA*), chloramphenicol resistance gene (*cmlA*,* floR*), sulfa resistance gene (*sul2*) and quinolone resistance gene (*gyrA*) were all 100%. The detection rates of quinolone resistance genes *parC* and *gyrB* were 57.14% and 61.90%, respectively. The detection rates of β-lactam resistance genes *bla*_*TEM*_ and *bla*_*DHA*_ were 47.62%. The detection rate of tetracycline resistance gene *tet(B)* was relatively low, only 4.76%. The comparison between the two serotypes showed that the probability of ST 967 *K. pneumoniae* isolate carrying *bla*_*TEM*_ was 8.33% (1/12), while the probability of ST 37 *K. pneumoniae* isolate carrying *bla*_*TEM*_ was 100% (9/9). There was no significant difference in the carrying of other resistance genes between the two serotypes.

**Fig. 9:**
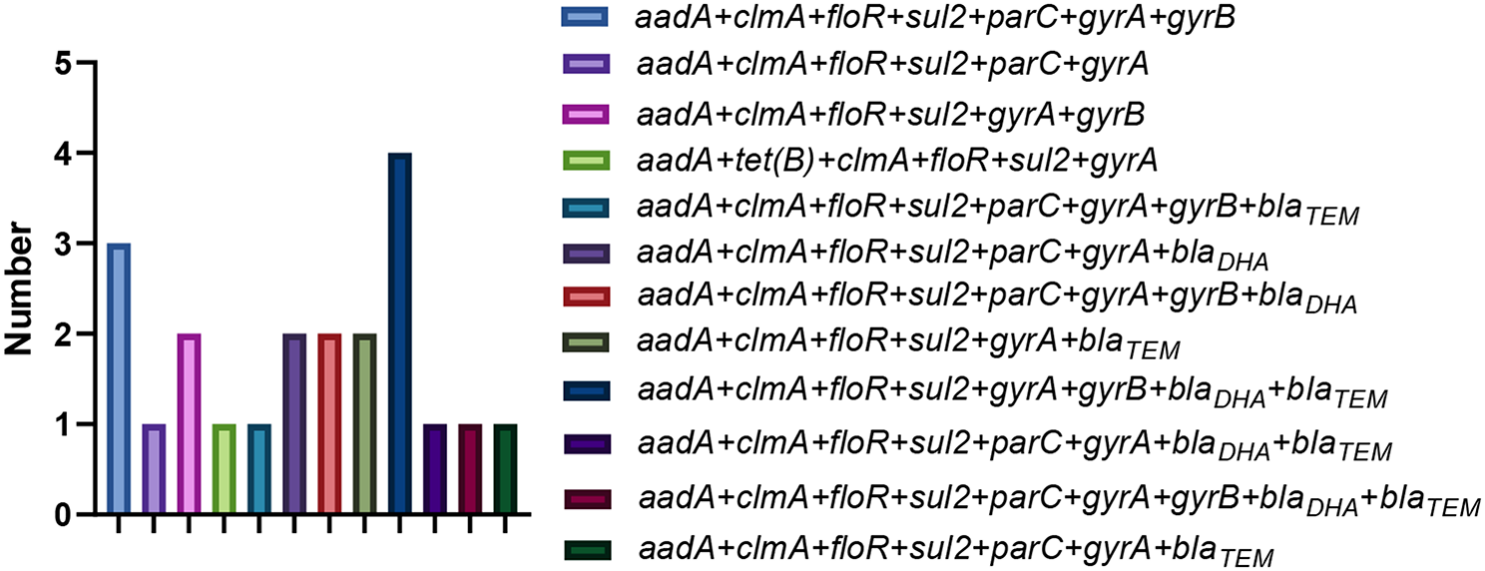
Antimicrobial Resistance gene combinations of different strains of *K. pneumoniae*

The analysis of drug resistance gene profiles of *K. pneumoniae* isolates showed that 12 kinds of drug resistance gene combinations were detected in 21 isolates. Among them, *aadA + clmA + floR + sul2 + parC + gyrA + gyrB + bla*_*DHA*_*+bla*_*TEM*_ was the combination with the largest number of drug-resistance genes, with a carrying rate of 4.76%. *aadA + clmA + floR + sul2 + gyrA + gyrB + bla*_*DHA*_*+bla*_*TEM*_ had the largest number of drug resistance gene combinations, with a carrying rate of 19.05% (Fig. 9). These results indicate that it is common for *K. pneumoniae* of pig origin to carry multiple drug resistance genes in Xinjiang.

As shown in Table 7, the drug-resistance phenotype and drug-resistance genotype of *K. pneumoniae* isolates were consistent. The detection rates of β-lactam, aminoglycoside and sulfonamide resistance genes were 100%. The coincidence rate of quinolone resistance was 95.24%. The compliance rate of tetracycline resistance was 4.76%.

**Table 7:** The detection rate of resistant genes of *K. pneumoniae* was consistent with the resistant phenotype

Types of Antibiotics	Number of drug-resistant strains/isolates	Number of resistant gene strains/strains	Detection rate of drug-resistant genes and conformity rate of drug resistance phenotypes/%
β-lactams	21	21	100%
Aminoglycosides	21	21	100%
Tetracyclines	21	1	4.76%
Sulfanilamide	21	21	100%
Quinolones	20	21	95.24%

#### Mucoid phenotyping

**Fig. 10:**
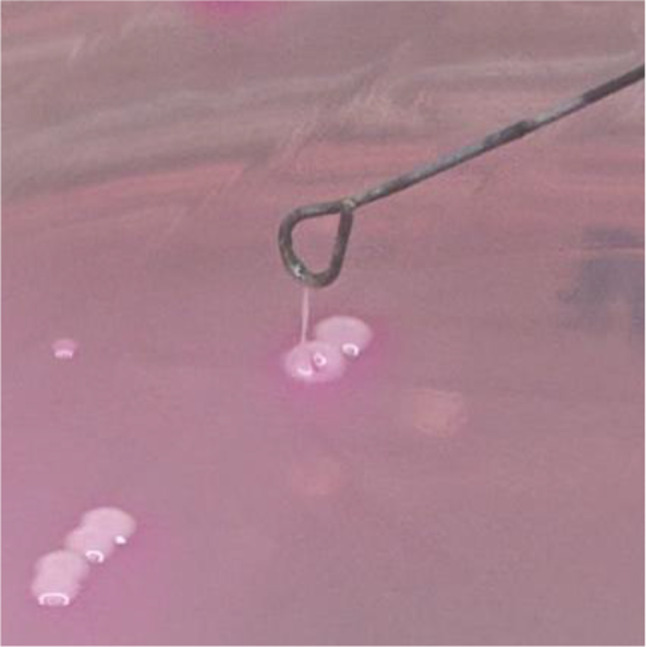
Drawing test result of some *Klebsiella pneumoniae* isolates

In the string test, none of the 21 strains of *K. pneumoniae* isolated showed a mucous thread length exceeding 5 mm, and all results were negative (Fig. 10), indicating that a high-mucus phenotype of *K. pneumoniae* was not detected.

#### Virulence gene detection

The detection of virulence genes of 21 *K. pneumoniae* isolates was shown in Fig. 8. A total of 8 virulence genes (*uge*,* wabG*,* entB*,* iroN*,* icuA*,* fimH*,* mrkD and ureA*) were detected. Among them, the highest detection rate was LPS related genes (*wabG*,* uge*,* entB*), *fimH*,* mrkD* and *ureA* related genes (*ureA*), accounting for 100%. The detection rates of *iucA* and *iroN* were 57.14% and 42.86%, respectively. Capsular-associated genes (*rmpA*,* magA*), ferrifer-associated genes (*kfu*) and allantoin genes (*allS*) were not detected. The comparison of virulence genes carried by two different serotypes showed that ST 967 *K. pneumoniae* isolates specifically carried virulence gene *icuA*, while most ST 37 *K. pneumoniae* isolates carried virulence gene *iroN*. There was no significant difference between the two serotypes in carrying other virulence genes.

**Fig. 11:**
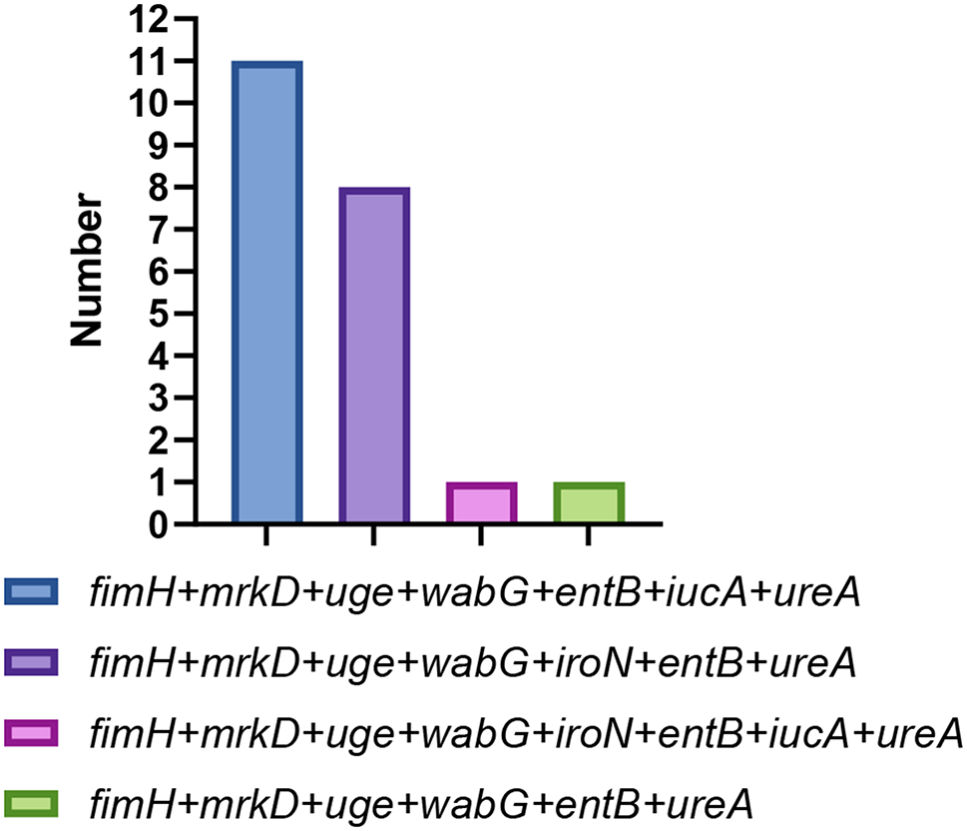
Virulence gene combinations of different strains of *K. pneumoniae*

According to the statistics of multiple virulence gene carrier, this isolate carried at least 6 virulence genes, of which *fimH + mrkD + uge + wabG + entB + iucA + ureA* had the largest virulence gene, accounting for 52.38%. The proportion of *fimH + mrkD + uge + wabG + entB + ureA* virulence gene and *fimH + mrkD + uge + wabG + entB + iucA + ureA + ironN* was the smallest, accounting for only 4.76% (Fig. 11). These results indicated that multiple virulence genes carried by *K. pneumoniae* from pigs were common in Xinjiang.

#### Mouse pathogenicity test

**Fig. 12:**
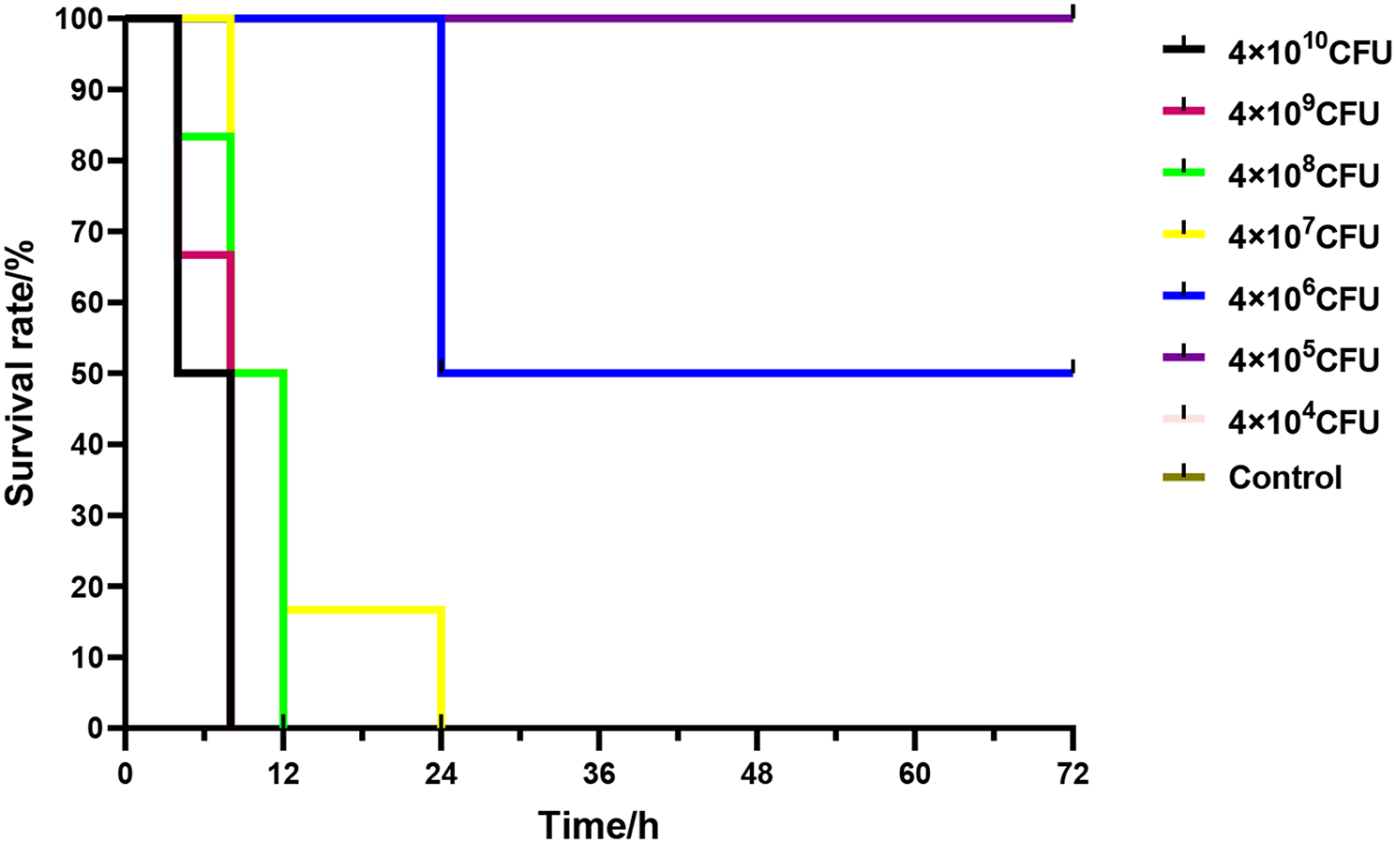
Survival curves of mice

**Fig. 13:**
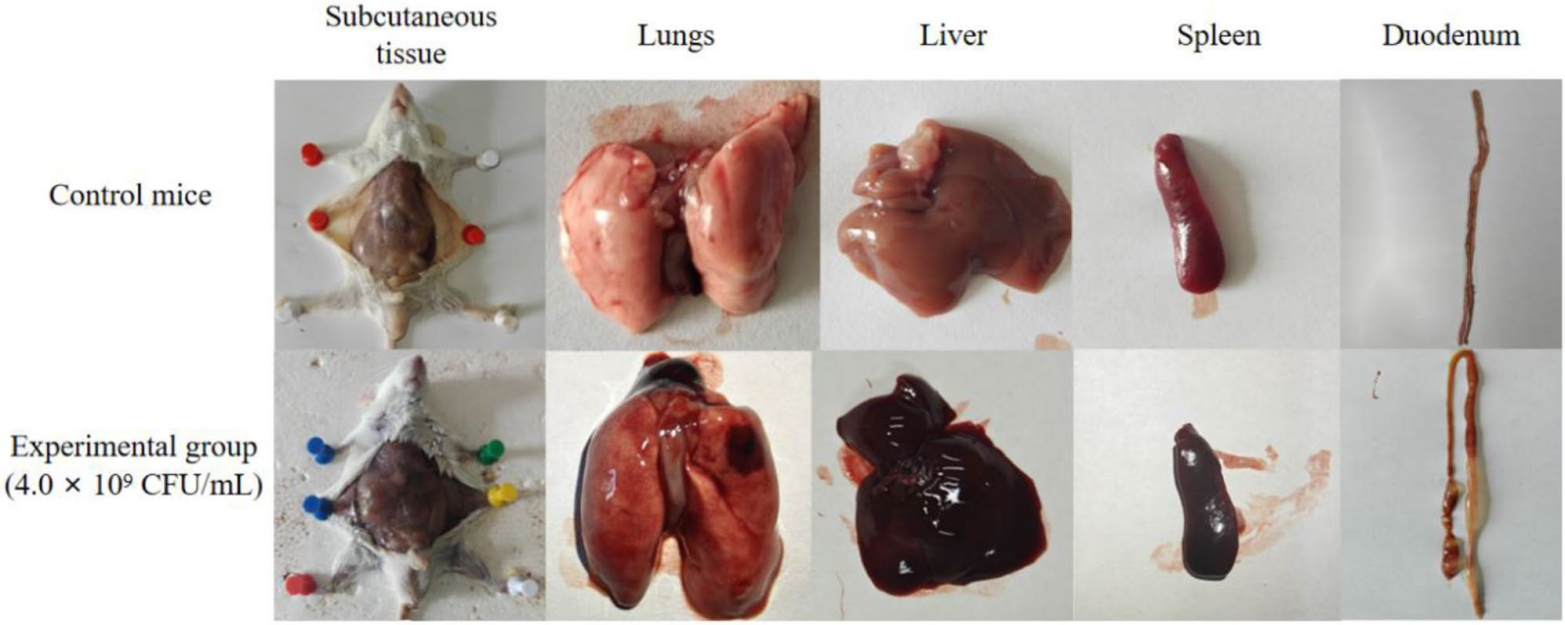
Pathological examination of mice

**Fig. 14:**
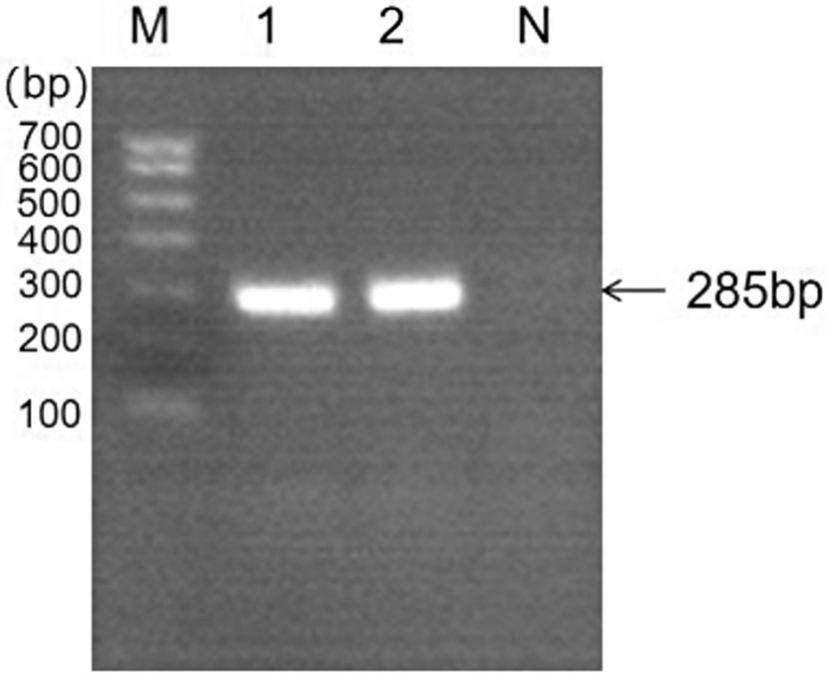
PCR amplification of *Khe* gene of bacteria isolated from various organs of infected mice. M: DL-700 Marker; 1:bacteria isolated from the lungs; 2: bacteria isolated from the livers N: negative control

**Fig. 15:**
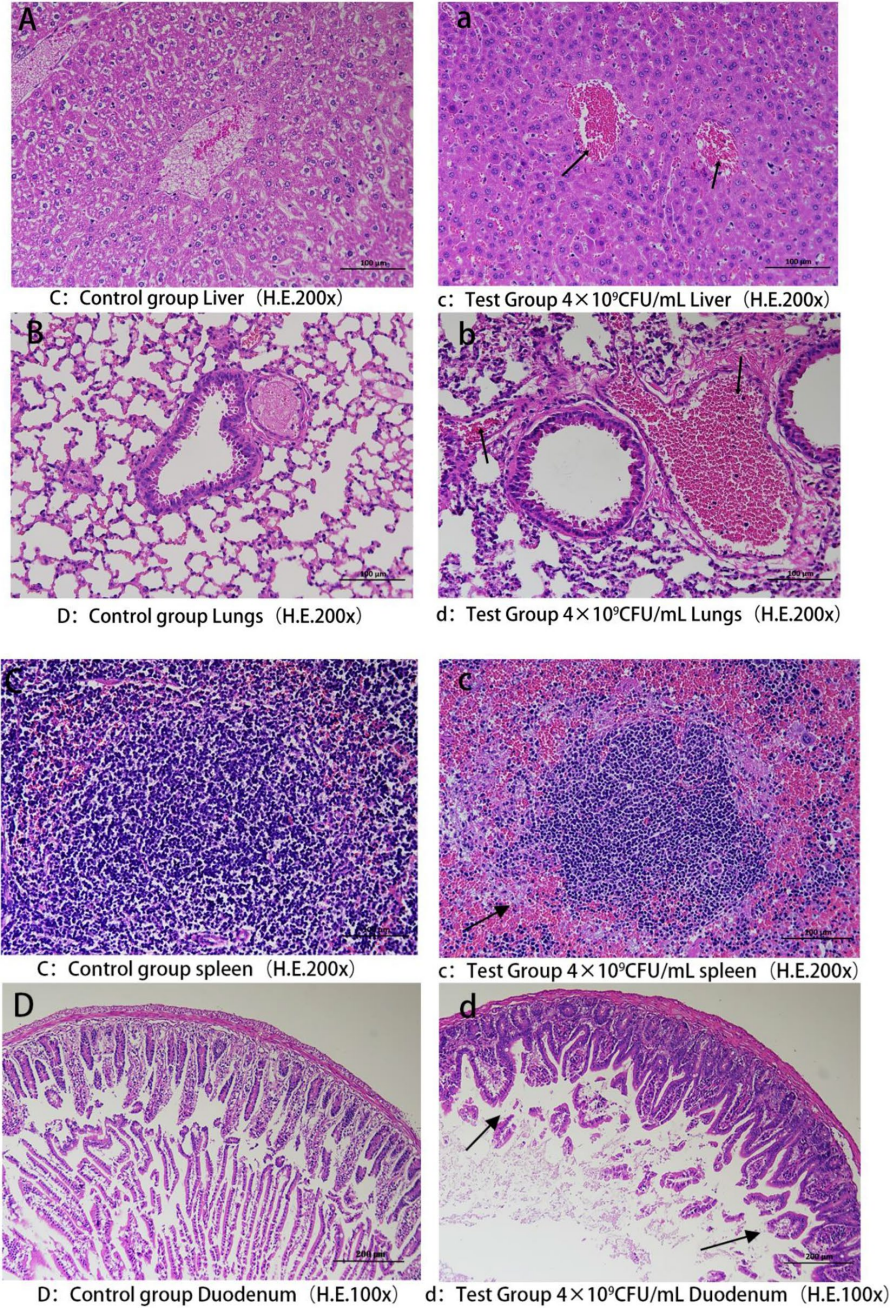
Histopathological observation of mice. *Note*** A**, **B**, **C**, **D** liver, lung, spleen and small intestine of mice in the control group; **a**, **b**, **c**, **d** liver, lung, spleen and small intestine of 4 × 10^9^ CFU/mL mice in the experimental group. *Note*** a** The arrow indicates a high concentration of red blood cells.; **b** The arrow indicates a high concentration of red blood cells; **c** The arrow points to an area with red blood cells and inflammatory exudate; **d** The arrow marks the site of small intestinal villi shedding

A comprehensive analysis was conducted on the drug resistance phenotypes, resistance genes, and virulence genes of 21 isolated strains, leading to the selection of the isolate KP-20 for a pathogenicity experiment in mice. The results revealed clinical symptoms in the experimental mice, including lethargy, reduced food and water intake, rapid breathing, and ruffled fur, beginning 4 h after intraperitoneal injection of the bacterial suspension. After 6 hours of infection, mice in the experimental group given 1.6 × 10^10^ CFU began to die, exhibiting symptoms such as hind limb convulsions and respiratory distress before death. By 24 h post-infection, all mice in four experimental groups receiving 1.6 × 10^10^, 1.6 × 10^9^, 1.6 × 10^8^, and 1.6 × 10^7^ CFU were dead, while three mice in the group receiving 1.6 × 10^6^ CFU survived; the remaining mice in other experimental groups were all alive. After 72 h, the surviving mice recovered to a normal state, with no abnormalities observed in the control group. The survival status of mice in each experimental group at different time points is shown in Fig. 12. Necropsy of the deceased mice revealed diffuse pulmonary hemorrhage, varying degrees of enlargement and congestion in the liver and spleen, localized distension in the small intestine, thinning of intestinal mucosa, and bleeding (Fig. 13). Bacteria were isolated from the lungs, liver, and spleen tissues of the dead mice, and PCR identification confirmed that the isolated strain matched the pathogenic strain (Fig. 14). Based on the death rates of the mice post-infection, the median lethal dose (LD_50_) of the isolate KP-20 was calculated to be 4.0 × 10^6^ CFU/mL. Pathological histological observations indicated disorganized liver cell arrangement, significant infiltration of red blood cells in the central vein, considerable hemorrhage and congestion in lung tissue, markedly reduced lymphocyte numbers in the white pulp of the spleen, and a significant increase in red blood cell numbers in the red pulp; the intestinal villi were found to be broken and sloughed off, with an influx of neutrophils in the submucosa, along with numerous villus fragments in the intestinal lumen (Fig. 15).

## Discussion

In 1882, *K. pneumoniae* was first isolated from the lungs of a patient who had died from pneumonia. Initially regarded as an opportunistic pathogen present in the natural environment, it did not receive widespread attention from society [29]. Currently, pneumonia caused by animal-derived *Klebsiella* bacteria typically demonstrates multidrug resistance and strong pathogenicity. The bacterium can acquire and spread antibiotic resistance genes through horizontal gene transfer mechanisms [30], it spreads widely among humans, the environment, and animals, posing a potential threat to public health and livestock farming [31]. In 2020, Dong [32] isolated 45 strains of *K. pneumoniae* from 123 samples of diseased tissue collected from various pig farms in the Cangzhou region, resulting in an isolation rate of 36.59%.In 2021, Fu [33] isolated a strain of *K. pneumoniae* from the diseased materials of a pig farm in Shaanxi. In 2022, Li [2] isolated a strain of *K. pneumoniae* (type ST-35) from the lung tissue of sick pigs at a large-scale pig farm in Guizhou.In 2022, Zuo [34] isolated three strains of *K. pneumoniae* from stool swab samples of diarrhea-prone Tibetan pigs from the Tibet region, with an isolation rate of 5%. In 2023, Xu [35] isolated 58 strains of *K. pneumoniae* from fecal swab samples collected from pig farms of varying scales in Fujian, achieving an isolation rate of 17.1% (58/340). In 2023, Hou [28] and others isolated three strains of *K. pneumoniae* from large-scale pig farms in the Xinjiang region, with an isolation rate of 4.55% (3/66) (one strain belonging to ST-967 type, one to ST-42 type, and one of unknown type). This research indicates that *K. pneumoniae* is prevalent in pig populations in China and has certain pathogenicity towards pigs.The study isolated 21 strains of *K. pneumoniae* from nasal swab samples of healthy pigs at another large-scale farm in Xinjiang, with an isolation rate of 42% (21/50). Mouse infection trials confirmed that the isolated strains exhibited moderate virulence in mice, consistent with existing research findings.

As of now, official MLST data shows that there are a total of 7,622 ST types of *K. pneumoniae* worldwide. ST 37 is primarily found in Europe, Asia, and North America, with most strains being of human origin. In contrast, ST 967 is mainly distributed in Asia and Europe, with some strains being of human origin and others of porcine origin. The porcine *K. pneumoniae* strains of ST 37 and ST 967 isolated in this experiment align with the known distribution patterns of *K. pneumoniae*. In 2017, China reported for the first time the isolation of ST 37 type *K. pneumoniae* from a newborn [36]. In 2024, Jing [37] and others isolated 13 strains of ST 37 type *K. pneumoniae* from the pig farm.This indicates that the ST 37 strain of *K. pneumoniae* is distributed among both the human population and pig populations in our country. In August 2019, researchers isolated the ST 967 strain of *K. pneumoniae* from a rehabilitated patient in Armenia [38]. So far, there has been little research on the ST 967 type of *K. pneumoniae*. In this experiment, 12 strains of ST 967 were isolated, all of which are multidrug-resistant. The ST 967 type is the predominant strain in this pig farm. Studies have indicated that both ST 37 and ST 967 types of *K. pneumoniae* pose a risk of transmission between humans and pigs, presenting a serious threat to public health safety.

Since the outbreak of African Swine Fever in 2018, the biosafety work of each farm is extremely strict, and disinfection is the top priority. The formation and spread of *K. pneumoniae* isolates in each enclosure is closely related to its strong biofilm formation ability. Li et al. [39] found that biofilm is an important mechanism of drug resistance of *K. pneumoniae*. The most important surface structures in the biofilm formation process of *K. pneumoniae* are type 3 pili and capsular polysaccharide. Fimbril mediates stable adhesion, while CPS ultimately affect biofilm structure and intercellular communication [10]. In this experiment, 21 strains of *K. pneumoniae* were isolated, and the detection rate of *fimH* and *mrkD* genes was 100%, but no capsular genes (*rmpA* and *magA*) were detected. Among the 21 strains, 10 strains had strong biofilm forming ability and 11 strains had medium biofilm forming ability, which was consistent with the multiple drug resistance shown in the drug susceptibility test. Meanwhile, the high detection rate of multiple drug resistance genes also indicated that the treatment of the bacteria after infection would be difficult. β-lactam, aminoglycosides, sulfonamides and quinolone resistance genes were detected in the KP isolates in this study to varying degrees, which is related to the clinical use of drugs in farms. Studies [40] have shown that resistance genes can be transmitted across strains and even between Gram-negative and Gram-positive bacteria. There are more than 400 acquired AMR genes in the genome of *K. pneumoniae*, twice the number of *E. coli*, and *K. pneumoniae* can acquire AMR genes from the environment [41].

With the intensive farming of animals, in order to reduce the economic losses caused by various bacterial diseases, antibacterial drugs are used in large quantities, resulting in the continuous emergence of drug-resistant strains. In this study, K-B method was used to detect the sensitivity of *K. pneumoniae* isolates to antibiotics. The results showed that 21 isolates showed strong resistance to β-lactam, aminoglycoside, tetracycline, sulfonamides, quinolones and other antibacterial drugs, and showed multiple drug resistance, and were only sensitive to carbapenem imipenem and polymyxins polymyxins B. These two drugs may be considered for later treatment of *K. pneumoniae* infection. Guan Zhongbin et al. isolated *K. pneumoniae* from 51 large-scale pig farms in Southwest China and showed high drug resistance to amoxicillin, ampicillin, kanamycin, gentamicin and other drugs, which was similar to the test results in this paper [42]. *K. pneumoniae* can synthesize β-lactamase and aminoglycoside passivase [43] and act on various parts of antibiotics, causing drugs to lose their original functions and thus developing resistance to corresponding antibiotics [44]. The resistance rate of *K. pneumoniae* isolates to penicillin G, amoxicillin, cefazolin, gentamicin and other drugs was 100%, which was consistent with the previous conclusions. The drug-resistant phenotype of bacteria is determined by the drug-resistant genotype, but the drug-resistant phenotype and drug-resistant genotype may not be completely consistent. The comparison between the drug-resistance phenotype and drug-resistance genotype of *K. pneumoniae* isolates showed that the compatibility rate of β-lactam, aminoglycoside, sulfonamides and quinolone resistance genes and drug-resistance phenotype of *K. pneumoniae* isolates was more than 95%, and the compatibility rate of tetracycline resistance genes and drug-resistance phenotype was only 4.76%. It is speculated that the cause may be the presence of undetected or unknown related resistance genes. In this study, the coincidence rate between β-lactam, aminoglycoside, drug resistance phenotype and drug resistance genes was similar to the results of Pang et al. [45]. In the process of breeding, a large amount of antibiotics, whether used for treatment or growth promotion, will activate relative drug resistance genes [46], which will contribute to the enhancement of strain resistance and increase the spread of these resistant strains to humans through the food chain or other transmission routes [43]. In this study, *K. pneumoniae* isolates developed strong resistance to 7 classes of 15 antibiotics, and were relatively sensitive to only 2 antibiotics. It is suggested that farms strictly control the use of antibiotics to prevent the emergence of more drug-resistant strains, and new antibacterial biologics can also be selected for prevention and control, so as to achieve accurate prevention and control and comprehensive treatment.

HvKP strains usually contain virulence genes such as *iucA*, *iroB*, *rmpA* and *rmpA2* [24]. Studies [47] have shown that iron uptake related genes (*iucA*) can increase the virulence of hvKP and is a key virulence characteristic of hvKP. In this study, the detection rate of *iucA* virulence gene in 21 *K. pneumoniae* isolates was 57.14%, and the positive strains were all ST 967. None of the 9 ST 37 isolates carried *iucA*, but a high probability of carrying *iroN* (88.89%). At the same time, the detection rate of *iucA* was higher than that of *K. pneumoniae* isolated by Min et al. [48] from various human samples from a hospital in Ningbo, China. Virulence genes *iucA* and *iroN* are related to the iron uptake system, while iro gene cluster mainly encodes salmonella, which is the C-glucosylated form of enterobacteriin. Studies have shown that more than 90% of *K. pneumoniae* that causes liver abscess can secrete salmonella [49]. It is speculated that the virulence of the two serotypes of *K. pneumoniae* isolates will be different, and further study will be conducted in the future. The expression of *entB* gene can produce more iron carriers, promote the development and growth of biofilm, and enhance the virulence of hvKP [50]. *fimH* and *mrkD* are adhesins encoding type I and type III fimilus, which can help *K. pneumoniae* break through the host defense system and are important virulence factors causing urinary tract infections. *wabG* is a Lipopolysaccharides (LPS) related gene encoding galactosyltransferase, which plays an important role in the synthesis of LPS, LPS can inhibit the phagocytosis of macrophages, thus leading to host susceptibility to infection [51]. *K. pneumoniae* toxic genes *mrkD*, *fimH*, *wabG* are conserved and can help bacteria grow and reproduce. In this study, the detection rates of *mrkD*, *fimH* and *wabG* of *K. pneumoniae* isolates were 100%, which was similar to the results of Zhu [52] and Zhang [53] et al.Studies have shown [54] that the virulence genes carried by *K. pneumoniae* are associated with resistance genes and antibiotic resistance phenotypes.Current research has found [55] that plasmids can transmit antibiotic resistance genes and virulence related genetic elements to *K. pneumoniae*. These plasmids can undergo frequent gene transcription, leading to plasmid fusion, resulting in an increasing number of highly virulent and carbapenem resistant *K. pneumoniae* strains.

In this study, the isolated strain KP-20 contained up to 8 virulence genes and 7 drug resistance genes, and had strong resistance to 13 antibiotics. The results of the mouse challenge test showed that the median lethal dose (LD50) of this strain to mice was 4.0 × 10^6^ CFU/mL, showing moderate pathogenicity and significantly stronger virulence than the pigeon derived *K. pneumoniae* isolated by Chen et al. [56] (LD50 = 4.68 × 10^9^ CFU/mL). After 8 h infection with high dose of KP-20, the mice died. The liver, lungs and spleen of the dead mice were bleeding and swollen to varying degrees, which fully demonstrated the pathogenicity of this strain. Although this strain was derived from healthy weaned piglets, it did not cause infection in the pig farm, which may be related to the strict implementation of various biosafety measures in the large-scale pig farm, but the test data showed that this strain has a certain potential threat to low-age piglets, pigs with low immunity and breeders.

## Conclusions

The porcine-derived *K. pneumoniae* isolated in this study has a strong ability to form biofilms, exhibits a strong phenomenon of multiple drug resistance, carries various drug resistance and virulence genes, has a moderate virulence to mice, and poses a potential threat to pig farming and public health safety. The use of polymyxin and imipenem antibiotics has a certain clearance effect on *K. pneumoniae*, and the use of β- lactam antibiotics should be strictly controlled to prevent other bacteria from developing antibiotic resistance except for *K. pneumoniae*. This study can provide a reference basis for the prevention and control of *K. pneumoniae* in pig farms.

## Electronic supplementary material

Below is the link to the electronic supplementary material.

Supplementary Material 1

Supplementary Material 2

## Data Availability

No datasets were generated or analysed during the current study.

## Abbreviations

*K. pneumoniae*: *Klebsiella pneumoniae*
MLST: Multilocus sequence typing
ST: Serotyping
PEN: Penicillin
AMX: Amoxicillin
CFB: Cefazolin
CTX: Cefotaxime
K: Kanamycin
GM: Gentamicin
ENR: Enrofloxacin
CIP: Ciprofloxacin
DX: Doxycycline
TMP: Trimethoprim-sulfamethoxazole
FON: Florfenicol
PB: Polymyxin B
AMP: Ampicillin
IPM: Imipenem
TCY: Tetracycline
CRO: Ceftriaxone
CA: Vancomycin

## References

[1] Huiwen Y, Mingyuan C, Hai-Zhao H et al. Isolation and identification of Klebsiella pneumoniae from dogs and analysis of drug resistance to cephalosporins. Heilongjiang Anim Husb Veterinary. 2023;(23):71–76+133–134.

[2] Li HM, Xiang W, Lu WB, Liu F, Lei LC, Zhang FX. Pathogenicity and drug sensitivity analysis of a porcine Klebsiella ST-35 pneumoniae strain. J Anim Husb Veterinary Sci. 2022;53(12):4356–66.

[3] Bidewell CA, Williamson SM, Rogers J, et al. Emergence of Klebsiella pneumoniae subspecies pneumoniae as a cause of septicaemia in pigs in England. PLoS ONE. 2018;13(2):e0191958. 29470491 10.1371/journal.pone.0191958 PMC5823397

[4] Bowring BG, Fahy VA, Morris A, Collins AM. An unusual culprit: Klebsiella pneumoniae causing septicaemia outbreaks in neonatal pigs. Vet Microbiol. 2017;203:267–70. 28619154 10.1016/j.vetmic.2017.03.018

[5] Zhao Feifei LI, Jie H, Ning XIE, Shiting ZENG, Zhenling. Drug resistance analysis of Klebsiella pneumoniae isolated from slaughterhouse. J Anim Husb Veterinary Sci. 2023;54(07):3044–53.

[6] Wang Liping SHAO, Chunhong F, Hui Z, Zheng TONG. Analysis of clinical and molecular characteristics of high virulence Klebsiella pneumoniae infection in abdominal cavity. Lab Med Clin. 2022;19(22):3050–4.

[7] Da Silva GJ, Mendonça N. Association between antimicrobial resistance and virulence in Escherichia coli. Virulence. 2012;3(1):18–28. 22286707 10.4161/viru.3.1.18382

[8] Niebaum J, Munakata Y. The development of relational reasoning: an Eyetracking Analysis of Strategy Use and Adaptation in children and adults performing Matrix Completion. Open Mind (Camb). 2023;7:197–220. 37416068 10.1162/opmi_a_00078 PMC10320822

[9] Choby JE, Howard-Anderson J, Weiss DS. Hypervirulent Klebsiella pneumoniae - clinical and molecular perspectives. J Intern Med. 2020;287(3):283–300. 31677303 10.1111/joim.13007 PMC7057273

[10] Clegg S, Murphy CN. Epidemiology and virulence of Klebsiella pneumoniae. Microbiol Spectr. 2016;4(1). 10.1128/microbiolspec.UTI-0005-2012 26999397

[11] Holden VI, Bachman MA. Diverging roles of bacterial siderophores during infection. Metallomics. 2015;7(6):986–95. 25745886 10.1039/c4mt00333k

[12] Albesa I, Eraso AJ, Frigerio CI, Lubetkin AM. [Outbreak of hospital infection, due to members of the Klebsielleae tribe, in an intensive care unit for infants]. Rev Argent Microbiol. 1980;12(2):39–43. 6755552

[13] Chang Junshuai D, Ruiying. Yang Shengyuan, etc. Isolation and identification of Enterococcus faecalis SL22 from pathogenic clinical mastitis. Chin J Anim Infect Dis. 2023;31(06):35–44.

[14] Brisse S, Passet V, Haugaard AB, et al. Wzi Gene sequencing, a rapid method for determination of capsular type for Klebsiella strains. J Clin Microbiol. 2013;51(12):4073–8. 24088853 10.1128/JCM.01924-13 PMC3838100

[15] M100; Performance Standards for Antimicrobial Susceptibility Testing. 32nd ed. Clinical and Laboratory Standards Institute: Wayne, PA, USA, 2022.

[16] Wei Xinchen. Isolation and identification of pathogenic Escherichia coli bacteriophages from pigs and evaluation of their killing effect on drug-resistant bacteria in the environment. M.D thesis, Huazhong Agricultural University, Wuhan, China, 2024.

[17] Li Jing L, Li T, Haixin, et al. Isolation, identification and detection of drug resistance and virulence genes of E. Coli from equine feces in some areas of Xinjiang. Chin Veterinary Sci. 2024;54(05):658–65.

[18] Candan ED, Aksöz N. Klebsiella pneumoniae: characteristics of carbapenem resistance and virulence factors. Acta Biochim Pol. 2015;62(4):867–74. 26637376 10.18388/abp.2015_1148

[19] Wu X, Liu J, Feng J, et al. Epidemiology, environmental risks, virulence, and Resistance determinants of Klebsiella pneumoniae from dairy cows in Hubei, China. Front Microbiol. 2022;13:858799. 35602033 10.3389/fmicb.2022.858799 PMC9117759

[20] Tabaran A, Mihaiu M, Tăbăran F, et al. First study on characterization of virulence and antibiotic resistance genes in verotoxigenic and enterotoxigenic E. Coli isolated from raw milk and unpasteurized traditional cheeses in Romania. Folia Microbiol (Praha). 2017;62(2):145–50. 27837411 10.1007/s12223-016-0481-8

[21] Fazel F, Jamshidi A, Khoramian B. Phenotypic and genotypic study on antimicrobial resistance patterns of E. Coli isolates from bovine mastitis. Microb Pathog. 2019;132:355–61. 31096003 10.1016/j.micpath.2019.05.018

[22] Kumabe A, Kenzaka T. String test of hypervirulent Klebsiella pneumonia. QJM. 2014;107(12):1053. 24890554 10.1093/qjmed/hcu124

[23] Alcántar-Curiel MD, Blackburn D, Saldaña Z, et al. Multi-functional analysis of Klebsiella pneumoniae fimbrial types in adherence and biofilm formation. Virulence. 2013;4(2):129–38. 23302788 10.4161/viru.22974 PMC3654611

[24] Russo TA, Olson R, Fang CT, et al. Identification of biomarkers for differentiation of Hypervirulent Klebsiella pneumoniae from classical K. pneumoniae. J Clin Microbiol. 2018;56(9):e00776–18. 29925642 10.1128/JCM.00776-18 PMC6113484

[25] Osman KM, Hassan HM, Orabi A, Abdelhafez AS. Phenotypic, antimicrobial susceptibility profile and virulence factors of Klebsiella pneumoniae isolated from buffalo and cow mastitic milk. Pathog Glob Health. 2014;108(4):191–9. 24915048 10.1179/2047773214Y.0000000141 PMC4069336

[26] Lan Y, Zhou M, Jian Z, Yan Q, Wang S, Liu W. Prevalence of pks gene cluster and characteristics of Klebsiella pneumoniae-induced bloodstream infections. J Clin Lab Anal. 2019;33(4):e22838. 30737883 10.1002/jcla.22838 PMC6528554

[27] Harada S, Doi Y. Hypervirulent Klebsiella pneumoniae: a call for Consensus definition and international collaboration. J Clin Microbiol. 2018;56(9):e00959–18. 29950337 10.1128/JCM.00959-18 PMC6113475

[28] Hou G, Ahmad S, Li Y, et al. Epidemiological, virulence, and Antibiotic Resistance Analysis of Klebsiella pneumoniae, a Major Source of Threat to Livestock and Poultry in some regions of Xinjiang, China. Anim (Basel). 2024;14(10):1433. 10.3390/ani14101433 PMC11117231 38791650

[29] Bengoechea JA, Sa Pessoa J. Klebsiella pneumoniae infection biology: living to counteract host defences. FEMS Microbiol Rev. 2019;43(2):123–44. 30452654 10.1093/femsre/fuy043 PMC6435446

[30] Hou GM, Zhou HQ, Yu XY, et al. Biological characteristics and genome analysis of a phage of multiple drug resistant Klebsiella pneumoniae from sheep. Chin Anim Husb Veterinary Med. 2024;51(05):2047–57.

[31] Giri S, Shekar M, Shetty AV, Shetty GPT. Antibiotic resistance and random amplified polymorphic DNA typing of Klebsiella pneumoniae isolated from clinical and water samples. Water Environ Res. 2021;93(11):2740–53. 34433233 10.1002/wer.1630

[32] Dong R, Sun X. Isolation of Klebsiella pneumoniae from pigs, drug resistance and detection of drug resistance genes in Cangzhou area. Chin J Veterinary Med. 2020;56(04):76–9.

[33] Fu Xia-Li ZHENG, Zi-Fang XIAO, Shu-Qi LI, Shuang. Isolation and identification of a strain of Klebsiella pneumoniae from swine. Chin Veterinary Sci. 2021;51(08):999–1006.

[34] Wei Z, Qi W, Qian Z et al. Isolation, identification and biological characteristics of Klebsiella pneumoniae from tibetan pigs. Heilongjiang Anim Husb Veterinary Med. 2022;(09):72–79+141.

[35] Xu Rui LAI, Huamin OU, Zhengyang et al. Isolation, identification and drug resistance detection of Klebsiella pneumoniae from pigs in Putian, Fujian Province. Heilongjiang Anim Husb Veterinary Med. 2023;(15):72–77+136.

[36] Li P, Wang M, Li X, et al. ST37 Klebsiella pneumoniae: development of carbapenem resistance in vivo during antimicrobial therapy in neonates. Future Microbiol. 2017;12:891–904. 28699768 10.2217/fmb-2016-0165

[37] Sheng XiJing. Molecular characterization of virulence and drug resistance of Klebsiella pneumoniae of porcine origin, 2024.

[38] Sheng J, Cave R, Ter-Stepanyan MM, et al. Whole-genome sequencing and Comparative Genomics Analysis of a newly emerged Multidrug-resistant Klebsiella pneumoniae isolate of ST967. Microbiol Spectr. 2023;11(3):e0401122. 37022188 10.1128/spectrum.04011-22 PMC10269624

[39] Li Naijing HE, Ping G, Xiu LIU, Yong LI, Shengqi. Effect of antibiotics on β-lactamase production in biofilm bacteria. Chin J Mod Med. 2006;(19):2928–30.

[40] Yang QE, Ma X, Zeng L, et al. Interphylum dissemination of NDM-5-positive plasmids in hospital wastewater from Fuzhou, China: a single-centre, culture-independent, plasmid transmission study. Lancet Microbe. 2024;5(1):e13–23. 38006896 10.1016/S2666-5247(23)00227-6

[41] Wyres KL, Holt KE. Klebsiella pneumoniae as a key trafficker of drug resistance genes from environmental to clinically important bacteria. Curr Opin Microbiol. 2018;45:131–9. 29723841 10.1016/j.mib.2018.04.004

[42] Zhongbin G, Hongning W, Bo Z et al. Isolation, identification and drug susceptibility test of Klebsiella pneumoniae infected pigs in large-scale pig farms. 2011;1.

[43] Ovejero CM, Escudero JA, Thomas-Lopez D, et al. Highly Tigecycline-resistant Klebsiella pneumoniae sequence type 11 (ST11) and ST147 isolates from Companion animals. Antimicrob Agents Chemother. 2017;61(6):e02640–16. 28396550 10.1128/AAC.02640-16 PMC5444156

[44] Bassetti M, Righi E, Carnelutti A, Graziano E, Russo A. Multidrug-resistant Klebsiella pneumoniae: challenges for treatment, prevention and infection control. Expert Rev Anti Infect Ther. 2018;16(10):749–61. 30207815 10.1080/14787210.2018.1522249

[45] Pang XM. Evaluation of drug resistance and virulence of Klebsiella pneumoniae from different host sources, 2024. 10.1038/s43856-025-01112-1 PMC12402274 40890500

[46] Jonas D, Reuter S, Klassen S, et al. Evaluation of the BD Phoenix CPO detect panel for prediction of Ambler class carbapenemases. Sci Rep. 2021;11(1):13150. 34162904 10.1038/s41598-021-92336-3 PMC8222379

[47] Shon AS, Bajwa RP, Russo TA. Hypervirulent (hypermucoviscous) Klebsiella pneumoniae: a new and dangerous breed. Virulence. 2013;4(2):107–18. 23302790 10.4161/viru.22718 PMC3654609

[48] Jiang M, Qiu X, Shui S, et al. Differences in molecular characteristics and expression of virulence genes in carbapenem-resistant and sensitive Klebsiella pneumoniae isolates in Ningbo, China. Front Microbiol. 2024;15:1356229. 38389531 10.3389/fmicb.2024.1356229 PMC10881320

[49] Lam M, Wick RR, Wyres KL, et al. Genetic diversity, mobilisation and spread of the yersiniabactin-encoding mobile element ICEKp in Klebsiella pneumoniae populations. Microb Genom. 2018;4(9):e000196. 29985125 10.1099/mgen.0.000196 PMC6202445

[50] Paauw A, Leverstein-van Hall MA, van Kessel KP, Verhoef J, Fluit AC. Yersiniabactin reduces the respiratory oxidative stress response of innate immune cells. PLoS ONE. 2009;4(12):e8240. 20041108 10.1371/journal.pone.0008240 PMC2795162

[51] Zhao Wei Y, Dong CHENG, Jianguo, et al. Advances in the study of Klebsiella pneumoniae virulence factors and their genomics. J Anhui Agricultural Univ. 2019;46(06):942–9.

[52] Zhu Lixia W, Hongbin ZHAO, Xiyan GAO, Guisheng SHI, Qiumei GAO, Guangping. Detection of some virulence genes, drug resistance genes and drug susceptibility of Klebsiella pneumoniae from fur animals. Chin Veterinary J. 2019;39(09):1744–52.

[53] Zhang Wenju L, Shaojie Z. Serotype identification, virulence gene and pathogenicity detection of Klebsiella pneumoniae from foxes in some areas of Hebei Province. Chin Veterinary J. 2020;40(04):735–9.

[54] Hou G, Ahmad S, Li Y et al. Epidemiological, virulence, and Antibiotic Resistance Analysis of Klebsiella pneumoniae, a Major Source of Threat to Livestock and Poultry in some regions of Xinjiang, China. Animals (Basel). 2024;14(10):1433. 10.3390/ani14101433 PMC11117231 38791650

[55] Yang X, Dong N, Chan EW, Zhang R, Chen S. Carbapenem Resistance-Encoding and virulence-encoding conjugative plasmids in Klebsiella pneumoniae. Trends Microbiol. 2021;29(1):65–83. 32448764 10.1016/j.tim.2020.04.012

[56] Chen Ruige L, Yunhui X, Wei, et al. Isolation, identification and biological characterization of Klebsiella pneumoniae ST443 from pigeon. Chin J Veterinary Med. 2024;60(05):46–55.

