# Supplementary figures and images for: Virulence characteristics and antibiotic resistance analysis of Klebsiella pneumoniae isolated from pig farms in Xinjiang, China: revealing potential zoonotic risks
Source: Porcine Health Manag. 2025 May 7;11:25. doi: 10.1186/s40813-025-00424-x (PMC12057239; doi:10.1186/s40813-025-00424-x)

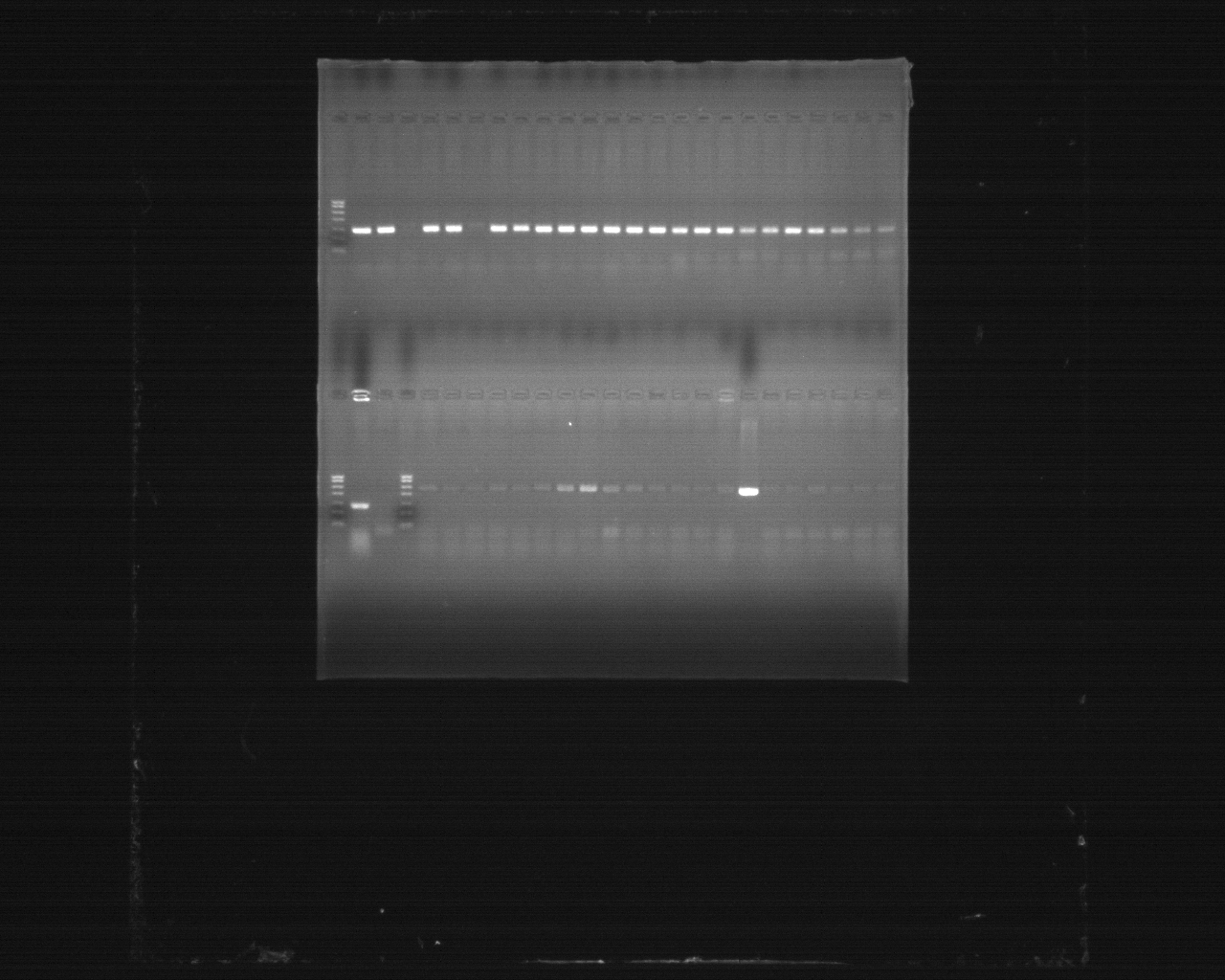

Supplement: Supplementary file 1 — Supplementary Material 1 [file 40813_2025_424_MOESM1_ESM.tif]

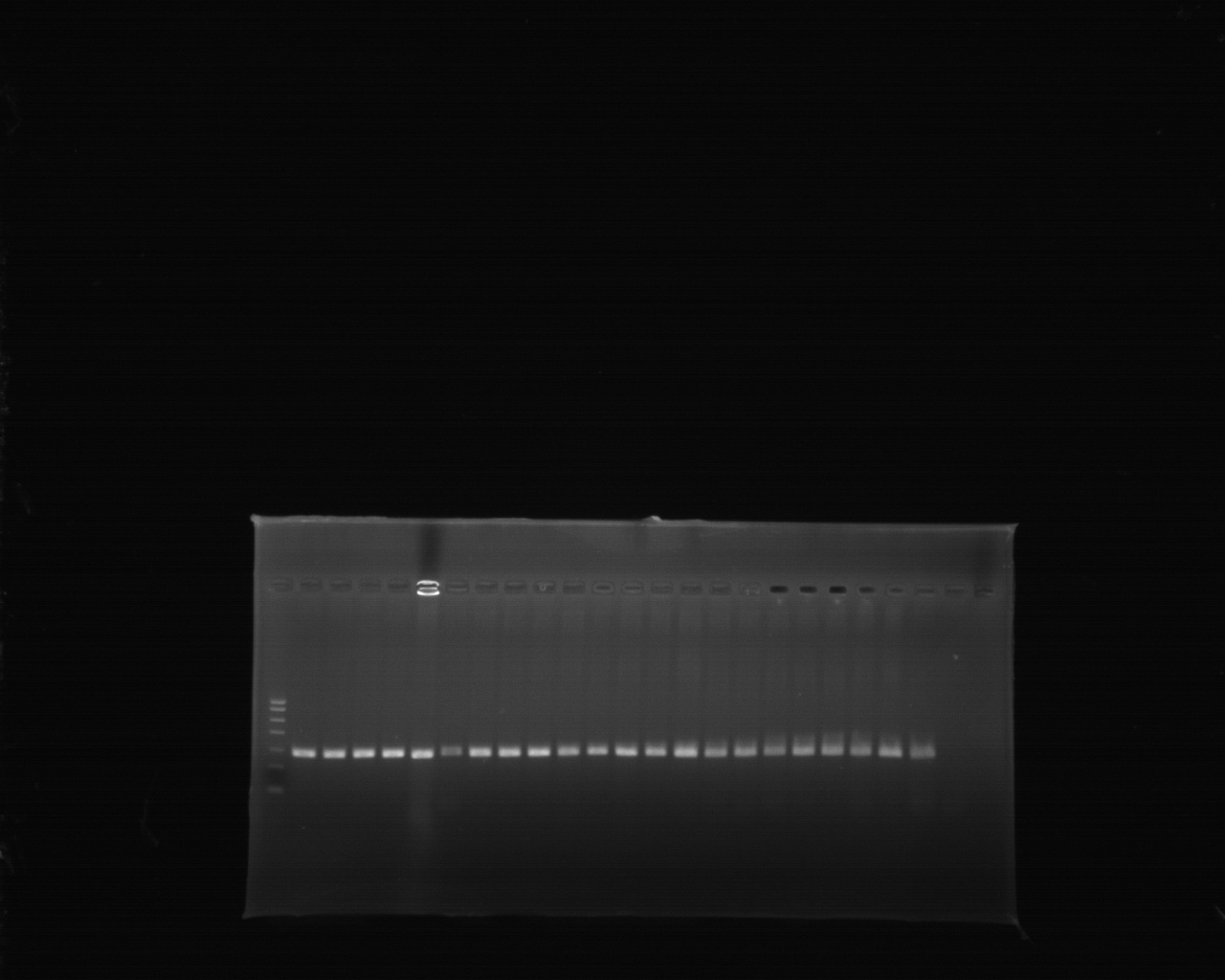

Supplement: Supplementary file 2 — Supplementary Material 2 [file 40813_2025_424_MOESM2_ESM.tif]
